# Mendelian Randomization Analysis of Genetic Proxies of Thiazide Diuretics and the Reduction of Kidney Stone Risk

**DOI:** 10.1001/jamanetworkopen.2023.43290

**Published:** 2023-11-14

**Authors:** Jefferson L. Triozzi, Ryan S. Hsi, Guanchao Wang, Elvis A. Akwo, Lee Wheless, Hua-Chang Chen, Ran Tao, T. Alp Ikizler, Cassianne Robinson-Cohen, Adriana M. Hung

**Affiliations:** 1 Division of Nephrology and Hypertension, Department of Medicine, Vanderbilt University Medical Center, Nashville, Tennessee; 2 Department of Urology, Vanderbilt University Medical Center, Nashville, Tennessee; 3 Department of Biostatistics, Vanderbilt University Medical Center, Nashville, Tennessee; 4 Department of Dermatology, Vanderbilt University Medical Center, Nashville, Tennessee; 5 Division of Epidemiology, Department of Medicine, Vanderbilt University Medical Center, Nashville, Tennessee; 6 Vanderbilt Genetics Institute, Vanderbilt University Medical Center, Nashville, Tennessee; 7 VA Tennessee Valley Healthcare System, Nashville, Tennessee

## Abstract

**Importance:**

Clinical trial data have called into question the efficacy of thiazide diuretics for the prevention of kidney stones.

**Objective:**

To identify whether there is an association between genetic proxies of thiazide diuretics and the risk of kidney stones.

**Design, Setting, and Participants:**

This genetic association study undertook a mendelian randomization analysis of derived exposures and outcomes from genome-wide association study summary statistics. Genetic proxies of thiazide diuretics were derived from the International Consortium for Blood Pressure. Kidney stone cases and controls were derived from the Million Veteran Program, UK Biobank, and the FinnGen study. These cross-sectional designs do not report a duration of follow-up. Data analysis was performed in May 2023.

**Exposure:**

Genetic proxies of thiazide diuretics were genetic variants in the thiazide-sensitive sodium chloride cotransporter gene associated with systolic blood pressure. Genetic proxies of β-blockers and systolic blood pressure served as negative controls.

**Main Outcomes and Measures:**

The main outcome was the odds of kidney stones. The secondary outcomes were serum laboratory values relevant to the treatment of kidney stones.

**Results:**

The main analysis included up to 1 079 657 individuals, including 50 832 kidney stone cases and 1 028 825 controls. In a meta-analysis of all cohorts, genetic proxies of thiazide diuretics were associated with a lower odds of kidney stones (OR, 0.85; 95% CI, 0.81-0.89; *P* < .001). Genetic proxies of β-blockers (OR, 1.02; 95% CI, 0.96-1.07; *P* = .52) and systolic blood pressure (OR, 1.00; 95% CI, 1.00-1.01; *P* = .49) were not associated with kidney stones. Genetic proxies of thiazide diuretics were associated with higher serum calcium (β [SE], 0.051 [0.0092]; *P* < .001) and total cholesterol (β [SE], 0.065 [0.015]; *P* < .001), but lower serum potassium (β [SE], −0.073 [0.022]; *P* < .001).

**Conclusions and Relevance:**

In this genetic association study, genetic proxies of thiazide diuretics were associated with reduced kidney stone risk. This finding reflects a drug effect over the course of a lifetime, unconstrained by the limited follow-up period of clinical trials.

## Key Points

**Question:**

Are thiazide diuretics associated with reduced risk of kidney stones?

**Findings:**

In this genetic association study of up to 1 079 657 adults, genetic proxies of thiazide diuretics were associated with a statistically significant 15% lower odds of kidney stones.

**Meaning:**

These results suggest that genetic proxies of thiazide diuretics estimate long-term drug effects; this finding supports the use of thiazide diuretics for kidney stone prevention.

## Teaser

This genetic association study including genome-wide association study summary statistics of US and European adults uses mendelian randomization analysis to examine the association of genetic proxies of thiazide diuretics with the risk of kidney stones.

## Introduction

Although kidney stones affect nearly 10% of the population worldwide, therapeutics are limited.^1,2^ Thiazide diuretics reduce the excretion of urinary calcium and are recommended by multiple clinical guidelines as a medical intervention for the prevention of calcium kidney stones.^3,4^ These recommendations are based on multiple clinical trials and meta-analyses, in which thiazides demonstrated a significant reduction in stone recurrence rates.^5,6,7^ However, the largest trial—the NOSTONE trial (Efficacy of Standard and Low Dose Hydrochlorothiazide Treatment in the Prevention of Recurrent Nephrolithiasis)—did not show a difference in composite symptomatic and radiographic stone recurrence among 416 patients with recurrent calcium-containing stones receiving various hydrochlorothiazide doses compared with placebo.^8,9^ The study’s ability to detect stone events may have been limited due to its short duration of follow-up. Additionally, the NOSTONE trial reported less reduction in urinary calcium than previous successful trials, suggesting that inadequate drug dosage or poor medication adherence may have contributed to its null treatment effect.^10,11,12^ However, a more extensive and long-term clinical trial would require significant resources.

Alternative approaches that use existing data sets are needed to understand the potential efficacy of thiazide diuretics, including large-scale data sets linked to genetic data suitable for analyzing drug effects. One such approach is mendelian randomization, which can investigate the association between naturally occurring genetic variation in drug targets and disease risk.^13^ By virtue of the random allocation of genetic variation at conception, this approach is less subject to unmeasured confounding or reverse causation biases that can occur in observational studies.^14^ Although it does not supersede the evidence of a randomized clinical trial, mendelian randomization provides an additional level of evidence for the preventive potential of thiazide diuretics in kidney stone disease.

In this study, genetic proxies of thiazide diuretics were derived from naturally occurring genetic variation in the thiazide-sensitive sodium chloride cotransporter gene. Kidney stone outcomes were derived from 3 biobanks. The objective was to use mendelian randomization to assess the association of genetic proxies of thiazide diuretics with the risk of kidney stones.

## Methods

### Overview

Mendelian randomization is a method for investigating potential causal relationships between exposures and disease outcomes.^15^ The first step is to identify genetic variants as instrumental variables that robustly associate with an exposure. These genetic variants can be located in or near the gene that encodes a drug target protein, and therefore instrumental variables can mimic drug effects.^16,17,18^ The second step is to assess the association between the instrumental variables and an outcome. This requires several assumptions, including that the genetic variants serving as instrumental variables are only associated with the exposure, have no common cause with the outcome, and only affect the outcome via the exposure.^19^

This study derived exposures and outcomes from genome-wide association studies (GWAS). Genetic proxies of thiazide diuretics and negative controls were derived from a GWAS of systolic blood pressure from the International Consortium for Blood Pressure (ICBP). Kidney stone outcomes were derived from GWAS in the Million Veteran Program (MVP), UK Biobank (UKB), and FinnGen study (FinnGen). The mendelian randomization association of genetic proxies of thiazide diuretics with kidney stones was estimated in each of these cohorts individually, and then combined in a random-effects meta-analysis (eFigure in Supplement 1).

This research adhered to the Strengthening the Reporting of Observational Studies in Epidemiology Using Mendelian Randomization (STROBE-MR) documentation.^51^ All analyses were conducted using GWAS summary statistics, which previously obtained ethical review board approvals; thus further authorization by institutional research boards of this secondary analysis was not required. Study protocols were not preregistered.

### Instrumental Variable Selection

To proxy thiazide diuretics, we identified genetic variants associated with systolic blood pressure at the *SLC12A3* gene and its 48 enhancer and promoter regions. The *SLC12A3* gene encodes the thiazide-sensitive sodium-chloride cotransporter located in the distal convoluted tubule of the kidney.^20^ This protein plays a critical role in sodium excretion and the maintenance of salt homeostasis. Thus, the sodium-chloride cotransporter function affects the regulation of blood pressure.^21^ Genetic proxies of β-blockers and systolic blood pressure were chosen as negative controls because they have no known effect on urinary calcium excretion or kidney stone risk and were used as negative controls in previous observational studies of kidney stone disease.^22^ To proxy β-blockers, we identified genetic variants associated with systolic blood pressure at the *ADRB1* gene and its 67 enhancer and promoter regions. The *ADRB1* gene encodes β1-adrenergic receptor, which is present in heart and kidney tissues and plays a role in cardiac contractility and renin release. To proxy the total effect of systolic blood pressure, we selected genetic variants associated with systolic blood pressure across the genome.

Genetic proxies for thiazides, β-blockers, and systolic blood pressure were selected from a GWAS of systolic blood pressure from the ICBP.^23^ Briefly, this global collaboration involved a fixed-effects inverse variance–weighted meta-analysis of several large-scale studies to identify common and rare genetic variants associated with blood pressure traits (757 601 total study participants). Details for the 77 independent studies and study-level genomic controls are provided in supplementary tables published by Evangelou et al.^23^ Summary statistics were downloaded from the OpenGWAS application programming interface (API).^24,25^ We identified genetic variants associated with systolic blood pressure from within drug target genes and their enhancer and promoter regions as determined from the GeneHancer integrated database.^26^ Significant associations had a Bonferroni level of significance based on the total number of genetic variants tested (ie, α < .05 divided by variants tested) (eTable 1 in Supplement 1).

We performed linkage disequilibrium clumping on significant genetic variants to select uncorrelated instrumental variables based on the 1000 Genomes Reference Panel using the European ancestry superpopulation as reference. This approach utilizes the PLINK clumping method, which prunes genetic variants in linkage disequilibrium within a specified 10 000 kb window, ultimately retaining the genetic variant with the lowest *P* value.^27^ The main analysis, negative controls, sensitivity analyses, heterogeneity tests, and pleiotropy tests used instrumental variables with a clumping threshold of *r^2^* = 0.4.^18^ To enhance the robustness of our findings, we used additional sensitivity analyses of the main analysis using instrumental variables with varying clumping thresholds from *r^2^* equaling 0.2, 0.1, 0.05, and 0.01, as done in previous drug-target mendelian randomization studies.^28^ The strength of instrumental variables was assessed using *F*-statistics, which are related to the variance in the phenotype explained by the genetic variants. *F*-statistics were estimated by the formula *F* = (β^2^ / standard error^2^).^29^ An *F* value above 10 was considered high instrument strength.^30^

### Main Analysis

Kidney stone outcomes were derived from GWAS summary statistics from 3 large biobanks—the MVP, UKB, and FinnGen. Kidney stones were defined using administrative and diagnosis codes. Kidney stones are a binary outcome based on the presence or absence of such codes and were not transformed. All 3 GWAS were minimally adjusted for age, sex, and 10 principal components of ancestry. These cross-sectional designs do not report a duration of follow-up. Data analysis was performed in May 2023.

The MVP GWAS was created as part of the genome-wide PheWAS project, a collaboration between the US Department of Veterans Affairs and the US Department of Energy^31^ (eMethods in Supplement 1). The case definition was urinary calculus as defined by phecode 594, and results were derived from European ancestry individuals defined by HARE (harmonized ancestry and race and ethnicity) (39 955 cases and 400 379 controls).^32^ Cases had at least 2 occurrences of the phecode on different days and controls had no occurrence of the phecode. The GWAS of urinary calculus was adjusted for age, sex, and first 10 principal components using a mixed model approach in SAIGE (Scalable and Accurate Implementation of Generalized mixed model).^33^ Results were filtered to remove variants with poor imputation quality (*r^2^* < 0.3) or that were very rare (minor allele count below 30).

The UKB is a biomedical database and research resource containing genetic, lifestyle, and health information from approximately half a million participants aged between 40 and 69 years in the UK, as described elsewhere.^34^ We obtained UKB data for individuals with European ancestry on the Pan-UKBB project phenotype trait “Calculus of Kidney Stone or Ureter” defined by *International Statistical Classification of Diseases and Related Health Problems, Tenth Revision *(*ICD-10*) code N20 (5530 cases, 415 001 controls). Summary statistics were downloaded from the Pan-UKBB project portal.^35^

The FinnGen includes analyses of the genome and health registry data of approximately half a million Finnish individuals, encompassing low frequency and high impact variants, as described elsewhere.^36^ We obtained FinnGen data from the IEU OpenGWAS project phenotype trait “Urolithiasis” defined by *ICD-10* codes N20, N21, and N23 (5347 cases, 213 445 controls). This reflects data freeze 5 (spring 2020), consisting of 218 792 individuals. Summary statistics were downloaded from the OpenGWAS API.^24,25^

### Secondary Outcomes

The secondary outcomes were serum laboratory values relevant to the treatment of kidney stones, including calcium, phosphorus, vitamin D, urate, albumin, alkaline phosphatase, potassium, cholesterol, glucose, and hemoglobin A_1c_. In clinical practice, these measurements are often used in the evaluation or follow-up of patients who receive thiazide diuretics for kidney stones.^37^ Thiazide diuretics reduce calcium excretion in the urine, which causes serum calcium levels to rise. They also cause extracellular volume contraction and consequently increase uric acid reabsorption in the proximal tubule of the kidney.^38^ Thiazides also affect insulin sensitivity and glucose metabolism leading to hyperglycemia. Hypercholesterolemia is affected by thiazide use, but the mechanism is not clearly understood.^39^ Finally, potassium levels in the serum fall from the diuretic-induced increased sodium delivery to the distal nephron as well as an indirect effect on aldosterone-mediated actions of the sodium–potassium pump in the collecting tubule of the kidney.^40^

The secondary outcomes were downloaded from the OpenGWAS API.^24,25^ The calcium, phosphorus, vitamin D, urate, albumin, alkaline phosphatase, potassium, cholesterol, glucose, and hemoglobin A_1c_ outcomes were derived from the Neale lab analysis of UKB round 2 GWAS. The GWAS was adjusted for age, sex, age squared, age × sex, age × sex squared, and the first 20 principal components, and outcomes were inverse rank-normal transformed. The potassium outcome was not available from that resource, so it was obtained from the BioBank Japan.^41,42^ The GWAS was adjusted for age, sex, and the first 10 principal components, and outcomes were also inverse rank-normal transformed.

### Statistical Analysis

#### Data Harmonization and Mendelian Randomization Analyses

The analyses were performed using the TwoSampleMR package in R. The associations of instrumental variables were extracted from the outcome GWAS using the outcome_data() function. For instrumental variables not present in the outcome data, a proxy in high linkage disequilibrium (*r^2^* > 0.8) would be substituted based on the 1000 genomes reference panel European population. Harmonization of genetic variants in the exposure and outcome data sets was performed using the harmonize_data() function. Ambiguous variants, where alleles do not correspond for the same genetic variant, were removed. Palindromic variants, where the alleles on the forward strand are equivalent to the reverse strand, were also removed unless the minor allele frequencies allowed us to infer which alleles were on the forward strand. The mendelian randomization inverse variance weighted effect estimate of drug proxies on outcomes was performed using the mr() function. The inverse-variance weighted effect estimate of drug proxies on kidney stones was calculated separately for MVP, UKB, and FinnGen, and then combined in a random-effects meta-analysis.^43^ The effect of drug proxies on all outcomes was inverted to reflect the negative relationship between blood pressure medications and blood pressure. An association was considered significant for 2-sided *P* < .05.

#### Sensitivity Analyses

Sensitivity analyses were performed using weighted median, weighted mode, and multiplicative random-effects inverse-variance weighted estimates at multiple clumping thresholds. The weighted median analysis estimates a causal effect while allowing for up to half of the single nucleotide polymorphisms to be invalid instrumental variables.^44^ The weighted mode determines causal effects assuming that the plurality of single nucleotide polymorphisms are valid instrumental variables, and has a low likelihood of inflating type 1 error.^45^ Lastly, examining the inverse-variance weighted at multiple clumping thresholds (with *r^2^* equaling 0.4, 0.2, 0.1, 0.05, and 0.01) allows for maximization of power at higher thresholds and maximizing validity at lower thresholds.^28^ The same sensitivity analyses were done for main outcome and the negative controls.

#### Heterogeneity and Pleiotropy Tests

The presence of heterogeneity or horizontal pleiotropy indicates potential violations of modeling assumptions, or that certain genetic variants are exerting a direct effect on the outcome not through the exposure.^46^ Heterogeneity was tested with Cochran Q and MR (mendelian randomization)-Egger Q statistics, which are expected to conform to a χ^2^ distribution with degrees of freedom equal to the number of genetic variants minus 1.^47^ Horizontal pleiotropy was tested with the MR-Egger intercept test, which suggests directional pleiotropy when the intercept of the MR-MR-Egger model is significantly different from zero, and the MR-PRESSO global test.^48,49^ If heterogeneity or pleiotropy were detected, the MR-PRESSO outlier test would be used to repeat the analyses after outlier removal.^50^ The same heterogeneity and pleiotropy tests were done for the main outcome and the negative controls.

#### Computing System

Analysis was performed using R version 3.3.3 (R Project in Statistical Computing) in the Vanderbilt Advanced Computing Center for Research and Education (ACCRE) computing environment. The TwoSampleMR package was used for the main analysis, secondary outcomes, sensitivity analyses, and heterogeneity and pleiotropy tests using the packages described in Methods. The ieugwasr package was used for clumping using the packages described in Methods. The MR-PRESSO package was used for MR-PRESSO heterogeneity and pleiotropy tests. The meta package was used for meta-analyses.

## Results

### Study Population

The study included up to 1 079 657 European ancestry individuals from the MVP (39 955 cases, 400 379 controls), UKB (5530 cases, 415 001 controls), and FinnGen (5347 cases, 213 445 controls) (Table). In the MVP, this included a majority men (92.6%) with a mean (SD) age of 64 (13.8) years, systolic blood pressure of 129 (15.6) mm Hg, diastolic blood pressure of 77.7 (10.2) mm Hg, and body mass index of 30.1 (5.6) (calculated as weight in kilograms divided by height in meters squared) with high prevalence of hypertension (60.1%) and diabetes (27.3%).

**Table.** Characteristics of the Study Population

Study	Case definition	*ICD-10* codes	Sample size, No.	Cases, No.	Age, mean (SD), y	Men, %	Hypertension, %	Diabetes, %
MVP^31^	Urinary calculus	N21.0, N21.1, N23, N20.0, N20.2, N21.8, N21.9, N20.9, N21, N20, N20.1, N22, Z87.442	440 334	39 955	64 (13.8)	93	60	27
UKB^56^	Calculus of kidney and ureter	N20	420 531	5530	56.5 (8.1)	46	36	14
FinnGen^57^	Urolithiasis	N20, N21, N22, N23	218 792	5347	59.8 (17.3)	44	26	16

Abbreviations: FinnGen, FinnGen study; *ICD-10*, *International Statistical Classification of Diseases and Related Health Problems, Tenth Revision*; MVP, Million Veteran Program; UKB, UK Biobank.

### Association of Genetic Proxies of Thiazide Diuretics With Kidney Stone Risk

Genetic proxies of thiazide diuretics, β-blockers, and systolic blood pressure were identified based on their association with systolic blood pressure in the ICBP (eTables 2-4 in Supplement 1). All instrumental variables demonstrated strong validity (*F*-statistic greater than 10) (eTable 5 in Supplement 1). The mendelian randomization effect of genetic proxies of thiazide diuretics on kidney stones was estimated in the MVP, UKB, and FinnGen individually (eTables 6-9 in Supplement 1) and then combined in a random-effects meta-analysis (eTable 10 in Supplement 1).

**Figure 1.**
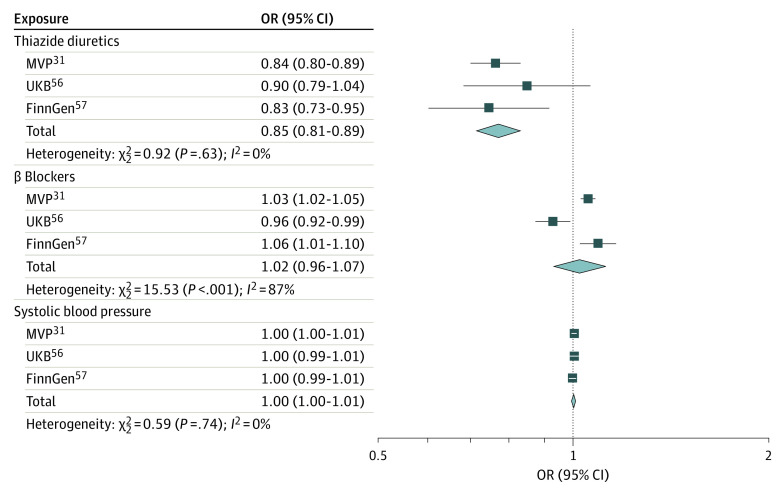
Association of Genetic Proxies of Thiazide Diuretics, β-Blockers, and Systolic Blood Pressure With Risk of Kidney Stones FinnGenn indicates the FinnGen study; MVP, Million Veteran Program; OR, odds ratio; UKB, UK Biobank.

In the meta-analysis, genetic proxies of thiazide diuretics demonstrated lower odds of kidney stones (OR, 0.85; 95% CI, 0.81-0.89; *P* < .001) (Figure 1). There was no evidence of heterogeneity (*P* for MVP Q = .67, *P* for MR-Egger Q = .60) or pleiotropy (*P* for MVP MR-Egger intercept = .55; *P* for MR-PRESSO global test = .78) (eTable 11 in Supplement 1). The effect estimate was consistently significant in the weighted median, weighted mode, and inverse variance weighted analyses when *r^2^* was set at 0.2, 0.1, 0.05, and 0.01 (*P* < .001 for all) (eTable 12 in Supplement 1).

Genetic proxies of β-blockers and systolic blood pressure served as negative controls. Genetic proxies of β-blockers did not have a significant effect in the meta-analysis (OR, 1.02; 95% CI, 0.96-1.07; *P* = .52). There was no evidence of heterogeneity (*P* for MVP Q = .67, *P* for MR-Egger Q = .61) or pleiotropy (*P* for MVP MR-Egger intercept = .69, *P* for MR-PRESSO global test = .72) (eTable 11 in Supplement 1). Similarly, genetic determinants of systolic blood pressure were not associated with the odds of kidney stones (OR, 1.00; 95% CI, 1.00-1.01; *P* = .49). There was evidence of heterogeneity (*P* for MVP Q < .001, *P* for MR-Egger Q < .001) and pleiotropy (*P* for MVP MR-Egger intercept = .81, *P* for MR-PRESSO global test < .001). However, no outliers were detected in the MR-PRESSO outlier test (eTable 12 in Supplement 1).

**Figure 2.**
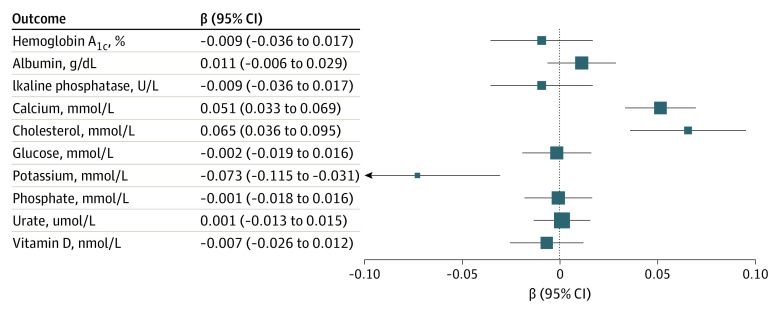
Association of Genetic Proxies of Thiazide Diuretics With Serum Laboratory Values The sizes of boxes indicate the precision of the effect estimate.

The association of genetic proxies of thiazide diuretics was tested against laboratory values related to the treatment of kidney stones (Figure 2). Genetic proxies of thiazide diuretics were associated with higher levels of serum calcium (β [SE], 0.051 [0.0093]; *P* < .001) and total cholesterol (β [SE], 0.065 [0.015]; *P* < .001) but lower levels of serum potassium (β [SE], −0.073 [0.022]; *P* < .001). There were no significant associations with hemoglobin A_1c_, albumin, alkaline phosphatase, glucose, phosphorus, urate, or vitamin D (eTable 13 in Supplement 1).

## Discussion

For over 3 decades, thiazide drugs have been the standard of care for the prevention of kidney stone recurrence.^3,4,52^ This practice was built upon observational data sets and multiple small clinical trials. However, the largest randomized clinical trial failed to find a protective effect of thiazides on kidney stone recurrence.^9^ Our mendelian randomization study provides further evidence that the action of thiazides may be sufficient to prevent kidney stone formation.

The use of mendelian randomization in this context offers several benefits. Genetic proxies may overcome potential confounding or reverse causation bias that may arise in studies of drug effects. For example, salt intake can increase urinary sodium and blunt the hypocalciuric effect of thiazide diuretics, but dietary habits are difficult to control in clinical studies.^53^ Mendelian randomization, on the other hand, is based on genetic variation assigned at birth and unaffected by environmental factors such as diet. Additionally, mendelian randomization provides a rapid and cost-effective means of investigating drug targets, whereas a large clinical trial would require immense effort and cost. Genetic proxies can approximate the effect of thiazide drugs over the course of a lifetime, as opposed to the confined duration of clinical trials.

The finding that genetic proxies of thiazide diuretics increase serum calcium while also reducing the risk of kidney stones is important. First, it supports the robustness of our instrumental variables, which appear to mimic the expected change in calcium seen in clinical practice. Prior reports estimate an increase of 0.8 mg/dL of serum calcium among thiazide users.^54^ Second, it supports the theory that modulation of calcium excretion through thiazide diuretics is a relevant mechanism for the reduced risk of kidney stones. While the exact mechanism is not completely understood, thiazides do appear to affect calcium absorption in the kidney and modulate uptake in bone.

### Limitations

It is important to acknowledge the limitations of the methods and data sources. The kidney stone phenotype was based on diagnosis codes that were not validated in the cohorts studied. However, analogous diagnosis codes have demonstrated a high positive predictive value for kidney stone events and treatments in other cohorts.^55^ This suggests that our outcome corresponds to clinical events rather than asymptomatic imaging findings or inconspicuous stone formation. The MVP cohort is a unique population consisting primarily of older, male veterans who may have different risk factors for urinary calculus than the general population. Additionally, the drug proxies obtained from the ICBP, the largest genetic study of blood pressure traits available, was derived from European ancestry individuals, which limits the generalizability of our results. The consortium also includes some overlap with the UKB, which may introduce bias into the estimate. Lastly, mendelian randomization relies on multiple assumptions, including the validity and independence of instrumental variables. While we demonstrate that the instrumental variables were related to the exposure based on robust *F*-statistics, we cannot definitively show that they were independent of the exposure-outcome relationship or the outcome itself. However, the results from the sensitivity analyses were consistent with our main analysis.

## Conclusions

In this genetic association study, genetic proxies of thiazide diuretics were associated with reduced kidney stone risk. In light of clinical trials that have challenged their efficacy, these findings may support the role of thiazide diuretics in the prevention of kidney stones.

## Notes

Supplement 1. **eFigure.** A Diagram of the Study Workflow **eTable 1.** Criteria for Instrumental Variables Selected From the International Consortium for Blood Pressure **eTable 2.** Instrumental Variables for Thiazide Diuretics From the International Consortium for Blood Pressure **eTable 3.** Instrumental Variables for Beta Blockers From the International Consortium for Blood Pressure **eTable 4.** Instrumental Variables for Systolic Blood Pressure From the International Consortium for Blood Pressure **eTable 5.** F-Statistics for Instrumental Variables **eTable 6.** Harmonization of Genetic Proxies of Thiazide Diuretics With Kidney Stone Risk in the Million Veteran Program, UK Biobank, and FinnGen Study **eTable 7.** Harmonization of Genetic Proxies of Beta Blockers With Kidney Stone Risk in the Million Veteran Program, UK Biobank, and FinnGen Study **eTable 8.** Harmonization of Genetic Proxies of Systolic Blood Pressure With Kidney Stone Risk in the Million Veteran Program, UK Biobank, and FinnGen Study **eTable 9.** The Inverse Variance-Weighted Effect of Genetic Proxies of Thiazide Diuretics, Beta Blockers, and Systolic Blood Pressure on Kidney Stones in the Million Veteran Program, UK Biobank, and FinnGen Study **eTable 10.** The Combined Random-Effects Model Meta-Analysis of Kidney Stone Risk in the Million Veteran Program, UK Biobank, and FinnGen Study **eTable 11.** Heterogeneity and Pleiotropy Testing for the Main Analysis and Negative Controls (Cochran’s Q, MR-Egger Q, MR-Egger Intercept, and MR-PRESSO Tests) **eTable 12.** Sensitivity Analyses for the Main Analysis and Negative Controls (Weighted Median, Weighted Mode, and Multiplicative Random-Effects Inverse Variance Weighted Effect Estimates at Multiple Clumping Thresholds) **eTable 13.** The Inverse Variance-Weighted Effect of Genetic Proxies of Thiazide Diuretics on Serum Laboratory Values **eMethods.** Genome-Wide PheWAS Methods From Million Veteran Program
eReferences.

Supplement 2.
Nonauthor Collaborators

Supplement 3.
Data Sharing Statement
