# Supplementary material for: Mendelian Randomization Analysis of Genetic Proxies of Thiazide Diuretics and the Reduction of Kidney Stone Risk
Source: JAMA Netw Open. 2023 Nov 14;6(11):e2343290. doi: 10.1001/jamanetworkopen.2023.43290 (PMC10646726; doi:10.1001/jamanetworkopen.2023.43290)
Supplement: Supplement 1. — eFigure. A Diagram of the Study Workflow eTable 1. Criteria for Instrumental Variables Selected From the International Consortium for Blood Pressure eTable 2. Instrumental Variables for Thiazide Diuretics From the International Consortium for Blood Pressure eTable 3. Instrumental Variables for Beta Blockers From the International Consortium for Blood Pressure eTable 4. Instrumental Variables for Systolic Blood Pressure From the International Consortium for Blood Pressure eTable 5. F-Statistics for Instrumental Variables eTable 6. Harmonization of Genetic Proxies of Thiazide Diuretics With Kidney Stone Risk in the Million Veteran Program, UK Biobank, and FinnGen Study eTable 7. Harmonization of Genetic Proxies of Beta Blockers With Kidney Stone Risk in the Million Veteran Program, UK Biobank, and FinnGen Study eTable 8. Harmonization of Genetic Proxies of Systolic Blood Pressure With Kidney Stone Risk in the Million Veteran Program, UK Biobank, and FinnGen Study eTable 9. The Inverse Variance-Weighted Effect of Genetic Proxies of Thiazide Diuretics, Beta Blockers, and Systolic Blood Pressure on Kidney Stones in the Million Veteran Program, UK Biobank, and FinnGen Study eTable 10. The Combined Random-Effects Model Meta-Analysis of Kidney Stone Risk in the Million Veteran Program, UK Biobank, and FinnGen Study eTable 11. Heterogeneity and Pleiotropy Testing for the Main Analysis and Negative Controls (Cochran’s Q, MR-Egger Q, MR-Egger Intercept, and MR-PRESSO Tests) eTable 12. Sensitivity Analyses for the Main Analysis and Negative Controls (Weighted Median, Weighted Mode, and Multiplicative Random-Effects Inverse Variance Weighted Effect Estimates at Multiple Clumping Thresholds) eTable 13. The Inverse Variance-Weighted Effect of Genetic Proxies of Thiazide Diuretics on Serum Laboratory Values eMethods. Genome-Wide PheWAS Methods From Million Veteran Program eReferences. [file jamanetwopen-e2343290-s001.pdf]

Supplemental Online Content

Triozzi JL, Hsi RS, Wang G, et al; for VA Million Veteran Program. Genetic proxies of thiazide diuretics reduce the risk of kidney stones in a Mendelian randomization analysis. *JAMA Netw Open.* 2023;6(11):e2343290. doi:10.1001/jamanetworkopen.2023.43290

eFigure 1. A Diagram of the Study Workflow

eTable 1. Criteria for Instrumental Variables Selected From the International Consortium for Blood Pressure

eTable 2. Instrumental Variables for Thiazide Diuretics From the International Consortium for Blood Pressure

eTable 3. Instrumental Variables for Beta Blockers From the International Consortium for Blood Pressure

eTable 4. Instrumental Variables for Systolic Blood Pressure From the International Consortium for Blood Pressure

eTable 5. F-Statistics for Instrumental Variables

eTable 6. Harmonization of Genetic Proxies of Thiazide Diuretics With Kidney Stone Risk in the Million Veteran Program, UK Biobank, and FinnGen Study

eTable 7. Harmonization of Genetic Proxies of Beta Blockers With Kidney Stone Risk in the Million Veteran Program, UK Biobank, and FinnGen Study

eTable 8. Harmonization of Genetic Proxies of Systolic Blood Pressure With Kidney Stone Risk in the Million Veteran Program, UK Biobank, and FinnGen Study

eTable 9. The Inverse Variance-Weighted Effect of Genetic Proxies of Thiazide Diuretics, Beta Blockers, and Systolic Blood Pressure on Kidney Stones in the Million Veteran Program, UK Biobank, and FinnGen Study

eTable 10. The Combined Random-Effects Model Meta-Analysis of Kidney Stone Risk in the Million Veteran Program, UK Biobank, and FinnGen Study

eTable 11. Heterogeneity and Pleiotropy Testing for the Main Analysis and Negative Controls (Cochran’s Q, MR-Egger Q, MR-Egger Intercept, and MR-PRESSO Tests)

eTable 12. Sensitivity Analyses for the Main Analysis and Negative Controls (Weighted Median, Weighted Mode, and Multiplicative Random-Effects Inverse Variance Weighted Effect Estimates at Multiple Clumping Thresholds)

© 2023 Triozzi JL et al. *JAMA Network Open.*

eTable 13. The Inverse Variance-Weighted Effect of Genetic Proxies of Thiazide Diuretics on Serum Laboratory Values

eTable 14. STROBE-MR Documentation

eAppendix. Genome-wide PheWAS Methods From Million Veteran Program

eReferences.

This supplemental material has been provided by the authors to give readers additional information about their work.

© 2023 Triozzi JL et al. *JAMA Network Open.*

eFigure 1. A diagram of the study workflow.

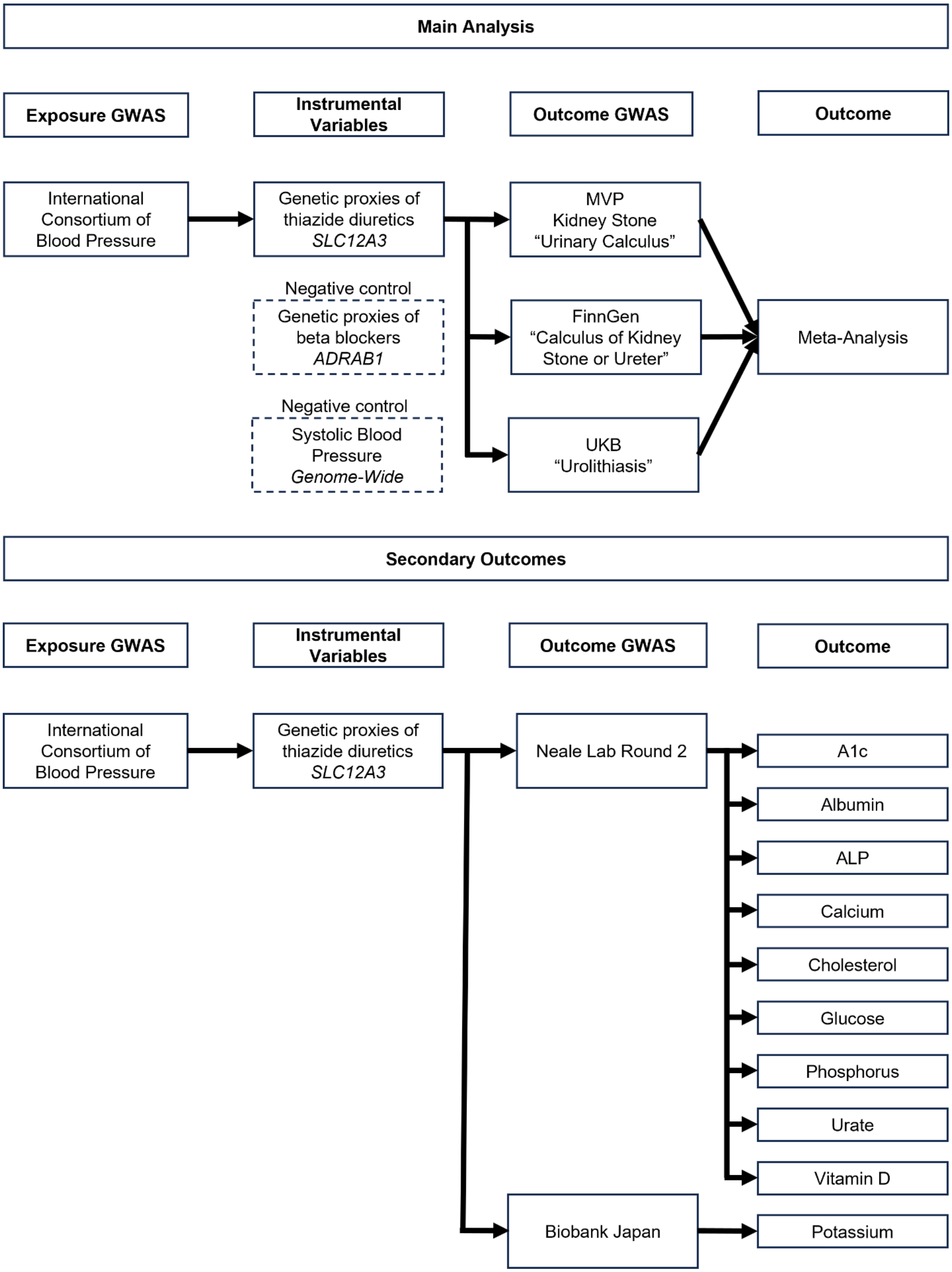

© 2023 Triozzi JL et al. *JAMA Network Open.*

eTable 1. Criteria for instrumental variables selected from the International Consortium for Blood Pressure.

Exp	Gene	Chr:BP (GRCh37/hg19)	Enhancer and promoter regions (N)	Total SNPs tested (N)	Bonferroni adjusted pval	SNPs at r^2 0.4 (N)	SNPs at r^2 0.2 (N)	SNPs at r^2 0.1 (N)	SNPs at r^2 0.05 (N)	SNPs at r^2 0.01 (N)
TZ	SLC12A3	16:56899119-56949762	48	455	1.10E-04	4	3	3	2	2
BB	ADRB1	10:115803806-115806667	67	250	2.00E-04	21	15	13	11	9
SBP	Genome-wide	NA	NA	NA	5E-08	NA	NA	NA	NA	461

Abbreviation: Exp, exposure; TZ, thiazide; BB, beta blocker; SBP, systolic blood pressure; Chr, chromosome; BP, base pair; SNP, single nucleotide polymorphism.

© 2023 Triozzi JL et al. *JAMA Network Open.*

eTable 2. Instrumental variables for thiazide diuretics from the International Consortium for Blood Pressure.

chr	position	beta	se	pval	rsid	ea	nea	eaf
16	56918125	-0.3527	0.0722	0.000001022	rs35797045	A	C	0.0493
16	56926195	-0.2054	0.0508	5.19194E-05	rs13306677	A	G	0.0994
16	56950210	0.1207	0.0304	7.16902E-05	rs9925265	A	G	0.4284
16	56820640	0.1752	0.0331	1.20601E-07	rs7500207	A	G	0.7131

Abbreviation: chr, chromosome; se, standard error; ea, effect allele; nea, non-effect allele; eaf, effect allele frequency.

© 2023 Triozzi JL et al. *JAMA Network Open.*

eTable 3. Instrumental variables for beta blockers from the International Consortium for Blood Pressure.

chr	position	beta	se	pval	rsid	ea	nea	eaf
10	115801253	0.3728	0.0303	7.39E-35	rs2429511	C	T	0.4799
10	115800294	0.3283	0.0552	2.66E-09	rs17875473	T	C	0.0871
10	115790006	-0.5804	0.0905	1.45E-10	rs79850079	A	G	0.0317
10	115792787	0.4557	0.0342	1.42E-40	rs740746	A	G	0.7318
10	115793237	0.1994	0.0463	1.65E-05	rs56012176	G	A	0.1234
10	115788094	0.2858	0.0458	4.23E-10	rs11196597	A	G	0.133
10	115823524	-0.4394	0.0482	7.88E-20	rs855715	T	G	0.1202
10	115781527	0.4249	0.0338	3.58E-36	rs2782980	C	T	0.7159
10	115831533	-0.3343	0.0406	1.69E-16	rs68122733	G	A	0.1718
10	115831756	0.1685	0.0353	1.76E-06	rs11196621	G	C	0.2466
10	115777836	0.1524	0.0313	1.12E-06	rs4256930	A	G	0.3643
10	115747742	0.1599	0.0308	2.06E-07	rs10787510	G	A	0.524
10	115748088	0.3692	0.0738	5.73E-07	rs17091184	T	G	0.0465
10	115713932	0.4322	0.1128	0.000128	rs139275657	G	A	0.0203
10	115718544	-0.5387	0.1099	9.46E-07	rs78793615	T	C	0.0213
10	115720673	0.6182	0.0738	5.33E-17	rs7086922	T	C	0.0452
10	115721364	0.2764	0.0324	1.36E-17	rs460718	G	A	0.6734
10	115926544	0.2127	0.0446	1.82E-06	rs17091398	C	T	0.1314
10	115704458	-0.3025	0.0732	3.57E-05	rs7894582	A	C	0.0466
10	115690539	-0.1727	0.0307	1.88E-08	rs11817866	T	A	0.4323
10	116438345	0.1254	0.0305	3.84E-05	rs10885595	T	C	0.5601

Abbreviation: chr, chromosome; se, standard error; ea, effect allele; nea, non-effect allele; eaf, effect allele frequency.

© 2023 Triozzi JL et al. *JAMA Network Open.*

eTable 4. Instrumental variables for the total effect of systolic blood pressure from the International Consortium for Blood Pressure.

chr	pos	beta	se	pval	rsid	ea	nea	eaf
1	1684169	-0.3385	0.0314	4.99689E-27	rs7796	G	C	0.4886
1	3327032	0.4183	0.0442	3.1369E-21	rs2493296	T	C	0.1425
1	10767902	-0.3799	0.0365	2.35396E-25	rs488834	T	C	0.7645
1	15810172	-0.3016	0.0377	1.17788E-15	rs75461554	T	C	0.2007
1	25366987	0.1935	0.0303	1.68298E-10	rs404100	T	C	0.4513
1	43765089	-0.2558	0.0313	2.84774E-16	rs1209384	G	A	0.6122
1	150572037	0.1796	0.0308	5.34306E-09	rs11585169	A	T	0.5773
1	169090660	-0.189	0.0307	7.21207E-10	rs12731646	T	C	0.409
1	184585182	0.1986	0.0306	8.99912E-11	rs4651224	T	C	0.4474
1	197297417	0.1807	0.0303	2.62398E-09	rs12042924	C	T	0.4716
1	207974818	-0.1938	0.0301	1.286E-10	rs2724377	G	A	0.4697
1	209970355	0.2294	0.0376	1.054E-09	rs7555285	C	G	0.8011
1	221265336	-0.2053	0.0328	3.80303E-10	rs72742507	T	C	0.2999
1	228199902	0.2874	0.0302	1.58708E-21	rs708117	A	G	0.5203
1	41865293	0.203	0.0313	8.91867E-11	rs11210029	G	A	0.3678
1	56606206	0.2965	0.0311	1.45011E-21	rs778124	A	G	0.3736
1	56979681	0.3181	0.0455	2.8622E-12	rs61772592	G	A	0.1255
1	67007389	0.2027	0.0304	2.68906E-11	rs12136922	A	G	0.4949
1	156129796	-0.2738	0.0461	2.97002E-09	rs76719272	T	C	0.1312
1	2164116	-0.1798	0.0307	4.71596E-09	rs263532	C	T	0.4245
1	6677064	-0.2191	0.032	7.43704E-12	rs10779795	G	A	0.3387
1	27407850	0.1992	0.0354	1.78098E-08	rs34079867	T	C	0.266
1	94051350	-0.2243	0.0361	5.45205E-10	rs7514579	C	A	0.2288
1	115827266	1.0997	0.1163	3.31513E-21	rs59980837	T	G	0.0178
1	217737629	0.274	0.0357	1.67803E-14	rs68085857	T	C	0.234
1	249155909	0.2965	0.0438	1.33291E-11	rs4926499	C	G	0.8263
1	11881441	-0.9115	0.041	1.6788E-109	rs6699618	G	C	0.1599
1	16348729	0.1782	0.0304	4.35101E-09	rs1889785	A	G	0.4552
1	59621911	0.1989	0.0318	3.85798E-10	rs12063372	A	G	0.3846
1	78555928	0.2028	0.0347	5.28506E-09	rs658780	G	T	0.2553
1	113044328	0.8211	0.0576	4.60999E-46	rs10776752	T	G	0.0809
1	180865798	-0.234	0.0312	6.32121E-14	rs10914124	C	T	0.3833
1	28838198	-0.2368	0.0331	8.33681E-13	rs2853736	G	T	0.3057
1	42364877	-0.3196	0.0304	8.33105E-26	rs1408945	T	G	0.4243
1	89242954	-0.3082	0.031	2.82488E-23	rs786923	T	C	0.6239
1	207211326	-0.1792	0.0307	5.12897E-09	rs11120093	T	C	0.4082
1	230849359	0.3736	0.0309	1.10408E-33	rs2493134	C	T	0.4071
1	243387788	0.1746	0.0311	1.935E-08	rs1565440	A	G	0.3752

© 2023 Triozzi JL et al. *JAMA Network Open.*

2	37517566	-0.3143	0.036	2.71519E-18	rs13420463	G	A	0.2266
2	43196694	-0.5893	0.0552	1.2939E-26	rs115262049	T	A	0.0868
2	65287896	-0.2927	0.0313	7.63484E-21	rs2249105	G	A	0.3679
2	66773469	0.1883	0.0307	8.79893E-10	rs10188003	T	C	0.393
2	68503044	0.1913	0.0326	4.18196E-09	rs6731373	A	G	0.3492
2	121996007	0.1769	0.0307	8.39305E-09	rs2580350	A	G	0.5609
2	174949358	0.1909	0.0309	6.41195E-10	rs11694601	G	A	0.4032
2	213188795	0.2018	0.0335	1.79602E-09	rs12694277	C	T	0.7054
2	218680529	-0.2836	0.0307	2.86814E-20	rs2161967	G	T	0.5721
2	242344695	-0.6115	0.0975	3.50502E-10	rs139354822	C	T	0.0296
2	19744462	0.2654	0.0304	2.2532E-18	rs17760259	C	T	0.4276
2	25187115	0.3266	0.0357	6.32995E-20	rs2384063	T	C	0.7607
2	26911745	-0.5411	0.0308	4.73042E-69	rs1275985	T	C	0.6133
2	145726621	0.2613	0.0355	1.79184E-13	rs55944332	G	A	0.2368
2	204085635	0.2575	0.0309	7.49031E-17	rs12693982	T	C	0.4024
2	40555733	-0.2124	0.0347	9.60395E-10	rs4952609	G	A	0.2561
2	135630498	-0.2274	0.0392	6.35097E-09	rs17257081	G	A	0.1935
2	164954174	0.5938	0.0353	1.77011E-63	rs268263	A	T	0.7498
2	191634958	-0.2889	0.0341	2.32488E-17	rs13412750	A	G	0.2708
2	230629138	-0.248	0.0424	5.15597E-09	rs1044822	T	C	0.1482
2	43397614	-0.2437	0.0315	1.01601E-14	rs12464602	A	G	0.6208
2	55779476	0.2522	0.0355	1.22687E-12	rs13016772	T	C	0.7651
2	69534650	-0.1737	0.0307	1.51702E-08	rs6732123	C	G	0.4174
2	73403040	0.1767	0.0302	4.98701E-09	rs4577304	C	T	0.4767
2	112744260	-0.2142	0.033	8.06121E-11	rs10207726	T	C	0.296
2	146989797	-0.1972	0.0321	7.68493E-10	rs62170470	C	T	0.3983
2	208521512	0.2942	0.0309	1.87586E-21	rs3845811	G	C	0.4339
2	86326717	-0.2413	0.0318	3.07893E-14	rs72847885	G	A	0.337
2	114083120	-0.2348	0.0364	1.12899E-10	rs6737318	G	A	0.2218
2	162278233	-0.3358	0.0521	1.153E-10	rs55732192	T	G	0.0947
2	177016728	0.2353	0.0324	4.01698E-13	rs34727427	C	T	0.3168
2	182981968	-0.2753	0.0363	3.33503E-14	rs1882212	G	A	0.2207
2	218779144	-0.1857	0.0318	5.29395E-09	rs3828282	G	C	0.5721
2	227185749	-0.2351	0.0306	1.62293E-14	rs10804330	C	T	0.4332
3	27562988	0.4473	0.0306	1.741E-48	rs2643826	T	C	0.4505
3	41107173	-0.2999	0.0432	3.80627E-12	rs6788984	G	A	0.1437
3	56771251	-0.2733	0.0324	3.09885E-17	rs3772219	C	A	0.3176
3	85656311	0.2579	0.0315	2.84381E-16	rs1375564	T	C	0.6395
3	121682388	0.1731	0.0302	9.94901E-09	rs12637573	G	A	0.5282
3	138101529	0.2654	0.047	1.65402E-08	rs1199330	G	A	0.1176
3	169534538	-0.2158	0.0348	5.63106E-10	rs4955575	C	A	0.2539

© 2023 Triozzi JL et al. *JAMA Network Open.*

3	185329756	0.2234	0.0325	6.14752E-12	rs13091418	G	C	0.3341
3	196228360	-0.2001	0.0318	3.223E-10	rs9869437	A	C	0.3523
3	20073193	-0.8664	0.139	4.54999E-10	rs189267552	A	T	0.0132
3	37598382	0.2597	0.0317	2.54976E-16	rs743395	T	C	0.3834
3	66422246	-0.1891	0.0331	1.10101E-08	rs7618284	C	G	0.3394
3	70920485	0.2199	0.0326	1.46285E-11	rs4499560	T	A	0.6829
3	74710462	-0.1727	0.0306	1.62301E-08	rs9857362	C	A	0.4709
3	141152017	0.4713	0.0651	4.64301E-13	rs9876694	T	C	0.0584
3	168697602	-0.4998	0.0487	1.02707E-24	rs3980686	T	G	0.1075
3	169096900	0.4124	0.0303	2.96825E-42	rs1290784	T	C	0.4483
3	183435713	-0.2371	0.0305	7.66655E-15	rs262986	A	G	0.4704
3	27704702	0.6445	0.1143	1.737E-08	rs68115553	G	A	0.0199
3	48108442	0.3793	0.0355	1.38899E-26	rs6771917	C	T	0.7523
3	53143901	-0.1891	0.0321	3.90301E-09	rs7615099	G	A	0.3325
3	133949366	0.3081	0.0308	1.59294E-23	rs9880098	A	G	0.3946
3	154680449	-0.4003	0.0504	2.08689E-15	rs79539362	C	T	0.1008
3	158212823	0.221	0.0339	7.30971E-11	rs6788907	A	G	0.2682
3	11495983	0.3231	0.0307	7.00971E-26	rs9848170	C	G	0.597
3	153729768	0.2301	0.0345	2.42493E-11	rs4408839	G	A	0.2567
3	14943965	-0.2901	0.0305	1.77582E-21	rs11925504	A	G	0.5721
3	41925398	0.2262	0.0412	4.14295E-08	rs1052501	T	C	0.8329
3	53562894	0.2774	0.0349	1.89583E-15	rs6445583	A	G	0.7465
3	124557643	-0.2736	0.0305	3.13184E-19	rs6438857	C	T	0.4226
3	169325621	0.1764	0.0302	5.22096E-09	rs2111557	T	C	0.4675
4	83830244	-0.2114	0.0351	1.73002E-09	rs60909079	C	G	0.2492
4	144051276	0.2243	0.0324	4.33711E-12	rs72719160	T	A	0.3171
4	18008232	0.2903	0.0343	2.86286E-17	rs2610990	G	A	0.7359
4	48713862	-0.2244	0.0304	1.59294E-13	rs62309747	A	G	0.4734
4	111408718	-0.3231	0.0466	3.90931E-12	rs1814951	A	G	0.8785
4	138464842	0.2537	0.0309	2.30622E-16	rs7439567	T	C	0.4106
4	145740898	0.2075	0.0358	6.84605E-09	rs2353940	C	T	0.2493
4	156639846	-0.3134	0.0313	1.23197E-23	rs12643599	G	A	0.3605
4	3451109	0.3171	0.0517	8.51491E-10	rs2498323	A	G	0.098
4	38387244	-0.2622	0.0303	5.10387E-18	rs2291434	T	G	0.5335
4	46595623	0.2329	0.0399	5.39299E-09	rs12511987	G	T	0.1774
4	81182554	0.8367	0.0334	2.5527E-138	rs12509595	C	T	0.2923
4	86719165	0.534	0.043	1.78115E-35	rs17010957	C	T	0.1463
4	148383424	0.2732	0.0434	3.04299E-10	rs73855810	A	G	0.1406
4	156402654	-0.3654	0.0304	2.43108E-33	rs7683728	T	C	0.5312
4	2668217	-0.2847	0.0327	3.17176E-18	rs1290933	A	C	0.6919
4	26812737	0.2651	0.0317	5.69508E-17	rs55924432	T	C	0.401

© 2023 Triozzi JL et al. *JAMA Network Open.*

4	89752913	-0.2937	0.0398	1.69083E-13	rs10028284	T	A	0.1816
4	103188709	-0.9086	0.0592	4.21891E-53	rs13107325	T	C	0.0739
4	108861082	0.1766	0.0318	2.72797E-08	rs1493132	C	T	0.3397
4	120555696	0.1973	0.0303	7.24102E-11	rs4834792	A	T	0.4796
4	169688000	-0.2115	0.0305	4.12382E-12	rs869396	A	C	0.4659
4	2246927	0.478	0.0786	1.181E-09	rs34535756	T	C	0.0394
4	54801228	-0.3789	0.0498	2.81968E-14	rs60991988	G	T	0.1069
4	63768826	-0.1778	0.0314	1.567E-08	rs13107261	A	G	0.3687
4	77414144	-0.2157	0.0302	9.2406E-13	rs10008637	C	T	0.4595
4	106911321	0.3628	0.043	3.35274E-17	rs11097909	C	T	0.8528
4	157678511	-0.3074	0.0429	7.61377E-13	rs17035181	G	T	0.1448
5	114428167	-0.2071	0.0326	2.22602E-10	rs11241313	T	C	0.3112
5	32831939	0.6382	0.0307	7.14003E-96	rs12656497	C	T	0.5966
5	50818437	-0.2048	0.0302	1.21004E-11	rs7722243	A	G	0.5043
5	61940569	0.455	0.059	1.26911E-14	rs12657950	T	C	0.0744
5	122470209	0.331	0.0309	1.05196E-26	rs1422279	T	C	0.3864
5	127411454	0.2024	0.0362	2.20699E-08	rs10045307	G	C	0.227
5	140086677	0.2318	0.0305	3.23892E-14	rs702395	T	C	0.4369
5	157474590	0.3629	0.0342	2.31686E-26	rs1957563	T	C	0.265
5	157938070	0.388	0.0445	2.94985E-18	rs13358657	G	A	0.1332
5	173301427	-0.2663	0.0333	1.20088E-15	rs3860770	A	G	0.2916
5	68007803	0.2479	0.0335	1.45312E-13	rs246973	T	C	0.2882
5	122835051	-0.2747	0.0319	8.06678E-18	rs9327297	G	C	0.3324
5	1279790	0.3098	0.0369	4.47301E-17	rs10069690	T	C	0.2582
5	15695987	-0.1985	0.0359	3.07199E-08	rs7725413	T	C	0.7699
5	33194751	0.2585	0.0332	6.41505E-15	rs10941043	G	T	0.2902
5	63831964	-0.2136	0.0347	7.58106E-10	rs6870654	C	T	0.2546
5	66280577	-0.2051	0.0344	2.355E-09	rs73103937	C	T	0.268
5	67678506	0.2246	0.0333	1.51217E-11	rs7703560	G	A	0.2998
5	87389027	-0.3143	0.0377	7.82168E-17	rs6452769	A	G	0.2053
5	96211594	-0.5233	0.0816	1.40101E-10	rs76443575	C	G	0.0359
5	157817634	-0.4727	0.0313	1.25285E-51	rs11960210	C	T	0.3755
5	179411477	-0.3303	0.0486	1.06905E-11	rs12153395	A	G	0.1147
5	361148	-0.1982	0.0323	8.11895E-10	rs4957026	G	A	0.6601
5	53276301	0.2643	0.0326	5.69246E-16	rs1664781	A	G	0.6925
5	55861464	0.2287	0.0387	3.304E-09	rs28650790	T	C	0.1891
5	97953719	0.1954	0.0324	1.656E-09	rs1871190	T	G	0.3349
5	127845030	0.3427	0.0307	7.11377E-29	rs6892983	A	C	0.4022
5	141726983	0.2418	0.0359	1.62293E-11	rs2913920	T	C	0.765
6	12903957	-0.2664	0.0312	1.30707E-17	rs9349379	G	A	0.407
6	31762844	0.4466	0.0309	2.66379E-47	rs67600122	T	G	0.4199

© 2023 Triozzi JL et al. *JAMA Network Open.*

6	79657391	0.2409	0.0303	1.76807E-15	rs1984195	A	G	0.4887
6	109013930	0.2657	0.0385	5.41876E-12	rs9486916	T	C	0.1979
6	25882678	0.5324	0.0499	1.42594E-26	rs79782817	T	G	0.1027
6	140692862	-0.1967	0.035	1.94299E-08	rs13204703	C	T	0.2489
6	151959945	0.1988	0.0347	1.034E-08	rs1293969	C	T	0.2516
6	163737476	0.2104	0.0374	1.81999E-08	rs12661036	C	T	0.225
6	1613686	-0.2164	0.0317	8.95571E-12	rs2745599	G	A	0.448
6	12295987	-0.1706	0.0309	3.51601E-08	rs1630736	T	C	0.465
6	39262535	0.2755	0.0458	1.76299E-09	rs2815063	A	C	0.1315
6	126228512	0.2106	0.0302	2.91206E-12	rs10782230	A	G	0.4845
6	127159982	0.5202	0.0305	3.65595E-65	rs9401913	A	G	0.4387
6	143200936	0.2134	0.0335	1.79999E-10	rs8180684	T	C	0.2896
6	22384718	-0.2369	0.0355	2.47913E-11	rs2744139	C	T	0.2376
6	30988017	0.4751	0.0747	1.98299E-10	rs12528975	A	G	0.0489
6	82235408	0.2196	0.0324	1.24796E-11	rs9361836	T	C	0.3172
6	117522156	0.1909	0.0305	3.74498E-10	rs961764	G	C	0.5746
6	134159976	-0.1844	0.0333	3.07298E-08	rs9285476	G	C	0.2929
6	7715689	0.1973	0.0301	5.58599E-11	rs1575290	T	C	0.4733
6	20686996	0.2281	0.0339	1.83696E-11	rs9368222	A	C	0.2688
6	43349215	0.3363	0.0321	1.17004E-25	rs7763558	A	G	0.3241
6	50683009	0.4287	0.0541	2.36483E-15	rs78648104	C	T	0.0925
6	97066242	0.3575	0.0385	1.57507E-20	rs6921291	T	C	0.1907
6	147713764	-0.201	0.0307	5.88166E-11	rs7765526	G	A	0.5367
6	151004770	-0.8085	0.0594	3.52209E-42	rs17080102	C	G	0.0694
6	159711515	-0.329	0.044	7.07946E-14	rs509833	G	A	0.8614
6	166176722	-0.4088	0.0593	5.63767E-12	rs7744902	A	G	0.0766
7	90297177	-0.1799	0.0322	2.39299E-08	rs67617547	G	C	0.3303
7	106414069	0.7507	0.0379	1.95794E-87	rs2392929	G	T	0.2027
7	130432469	-0.2422	0.0303	1.37309E-15	rs34072724	A	G	0.4889
7	4669949	0.2382	0.0392	1.19699E-09	rs73049928	G	A	0.1939
7	18543250	0.1859	0.0319	5.38902E-09	rs3807925	G	A	0.3504
7	47548893	0.2151	0.035	7.88297E-10	rs12668436	C	T	0.2459
7	130973495	0.2694	0.031	3.76184E-18	rs35680304	T	C	0.5929
7	2529623	-0.302	0.0327	2.45584E-20	rs10282122	T	C	0.6684
7	1966831	0.2344	0.031	4.21794E-14	rs200700882	G	A	0.4019
7	19039605	0.3222	0.038	2.33507E-17	rs28688791	C	T	0.1982
7	24735004	-0.2641	0.0477	3.179E-08	rs112509803	C	G	0.1138
7	131361319	-0.2064	0.0345	2.197E-09	rs6957161	G	A	0.7382
7	149474622	0.3616	0.0623	6.60404E-09	rs73727605	A	G	0.0663
7	150690176	0.664	0.0575	8.46058E-31	rs3918226	T	C	0.0811
7	151413194	0.3831	0.034	1.60398E-29	rs10224210	C	T	0.2789

© 2023 Triozzi JL et al. *JAMA Network Open.*

7	27245893	0.91	0.0577	5.29054E-56	rs3735533	C	T	0.9257
7	27328187	0.5304	0.0497	1.42988E-26	rs6961048	G	C	0.104
7	46008110	-0.3213	0.0312	6.62217E-25	rs11977526	A	G	0.4009
7	77572461	0.2025	0.0339	2.28402E-09	rs848445	C	T	0.7149
7	92237426	-0.3231	0.0345	7.39265E-21	rs42032	A	G	0.2641
7	131321010	0.5885	0.0839	2.34801E-12	rs75672964	T	C	0.0418
7	155744303	-0.206	0.0311	3.60496E-11	rs1870735	G	C	0.5469
8	25889446	-0.4222	0.0348	6.67421E-34	rs7821832	G	T	0.2553
8	38130025	0.2966	0.0358	1.20393E-16	rs1906672	A	G	0.2319
8	92793786	0.1844	0.0321	9.2151E-09	rs4734868	G	A	0.3303
8	141057641	-0.2201	0.0312	1.87413E-12	rs4440615	A	G	0.6321
8	1710404	0.22	0.0336	6.2273E-11	rs4876133	C	G	0.711
8	77681097	-0.2975	0.0498	2.28402E-09	rs9918876	A	C	0.1037
8	81386066	-0.4623	0.0848	4.96798E-08	rs148401029	A	C	0.0352
8	120358445	-0.3454	0.0392	1.28204E-18	rs2470004	T	C	0.8175
8	10640065	-0.3794	0.0307	5.19159E-35	rs1821002	G	C	0.5892
8	23402482	0.2662	0.0363	2.40381E-13	rs7844887	A	G	0.2208
8	26043622	0.3467	0.0485	8.37722E-13	rs77375686	G	A	0.1117
8	64501744	-0.2507	0.0317	2.42326E-15	rs2354862	C	A	0.3593
8	82828857	-0.2066	0.0306	1.41808E-11	rs62512914	G	A	0.4152
8	101674751	-0.2073	0.0307	1.344E-11	rs1786345	C	A	0.4338
8	126513197	0.2132	0.0329	9.62498E-11	rs6986368	T	A	0.3274
8	129386613	-0.1911	0.0314	1.17101E-09	rs4260863	G	C	0.3837
8	51947549	0.3431	0.0403	1.61399E-17	rs4873492	T	C	0.1724
8	95278307	0.3124	0.0454	5.96623E-12	rs34917849	C	G	0.1268
8	129170126	-0.1805	0.032	1.60901E-08	rs2608029	G	C	0.3347
8	143991858	-0.2753	0.0305	1.80593E-19	rs7463212	A	T	0.5445
8	68920135	0.2127	0.033	1.128E-10	rs13253358	T	C	0.2979
8	76878957	-0.2601	0.0306	1.87111E-17	rs2126474	T	G	0.4125
8	105921209	0.4005	0.0727	3.67799E-08	rs79069610	C	T	0.05
8	105966258	-0.4619	0.0507	8.81455E-20	rs35783704	A	G	0.1042
8	135616959	0.2325	0.0301	1.21088E-14	rs7012866	G	T	0.5009
8	141812374	0.2268	0.0303	7.3536E-14	rs4961293	T	C	0.4513
9	22942770	-0.2048	0.0343	2.47201E-09	rs9886665	C	T	0.7329
9	77239540	0.2173	0.0388	2.16501E-08	rs1410222	T	C	0.8166
9	128153616	-0.2449	0.0303	6.31393E-16	rs3104552	C	T	0.4393
9	9350706	-0.2203	0.0314	2.31686E-12	rs1332813	C	T	0.6486
9	113249071	0.7606	0.0827	3.82825E-20	rs10980408	C	T	0.0359
9	753648	0.2382	0.0385	5.87503E-10	rs60191654	G	A	0.1882
9	35906471	-0.2974	0.0397	7.12853E-14	rs76452347	T	C	0.205
9	116670743	-0.1869	0.0299	4.00701E-10	rs7026176	T	G	0.5118

© 2023 Triozzi JL et al. *JAMA Network Open.*

9	125657099	-0.3009	0.0453	3.12968E-11	rs4838021	T	C	0.1289
9	4117713	0.1689	0.0303	2.43798E-08	rs927315	T	C	0.4713
9	34223553	-0.2035	0.03	1.09396E-11	rs4553000	T	C	0.5141
9	95201540	-0.1862	0.0313	2.54501E-09	rs7045409	A	T	0.3669
9	123516572	-0.223	0.0308	4.70652E-13	rs34025993	G	A	0.586
9	136522274	-0.5547	0.0611	1.18304E-19	rs6271	T	C	0.0735
9	139520789	-0.2135	0.0322	3.53671E-11	rs11145807	G	A	0.5943
10	45377839	-0.2256	0.0338	2.60976E-11	rs4284362	A	C	0.7181
10	60365755	0.1794	0.0305	4.03497E-09	rs4245599	G	A	0.5416
10	82136664	-0.203	0.0302	1.88018E-11	rs10749572	T	G	0.5444
10	95899706	-0.2702	0.0316	1.14604E-17	rs2689690	T	C	0.3678
10	4124568	-0.4164	0.0573	3.61327E-13	rs11252324	T	G	0.0771
10	18727959	-0.6327	0.0337	1.08293E-78	rs12258967	G	C	0.2953
10	48411796	-0.3016	0.0497	1.279E-09	rs34130368	T	G	0.117
10	63499951	-0.716	0.0426	2.10184E-63	rs57946343	C	T	0.1473
10	94441507	0.3569	0.0597	2.28802E-09	rs111866816	T	C	0.0709
10	104906211	-1.0995	0.055	7.73749E-89	rs11191580	C	T	0.0824
10	32289986	0.2135	0.0387	3.58402E-08	rs12264186	T	C	0.1871
10	75409877	0.4394	0.0432	2.79705E-24	rs2177843	T	C	0.1505
10	102553647	0.6846	0.048	3.49704E-46	rs1006545	T	G	0.8872
10	115792787	0.4557	0.0342	1.42102E-40	rs740746	A	G	0.7318
10	133773019	-0.2144	0.0322	2.93833E-11	rs7912283	A	G	0.6468
10	114808902	0.2358	0.0335	1.93821E-12	rs12255372	T	G	0.2883
10	122968964	0.3024	0.0326	1.54703E-20	rs11592107	A	G	0.3096
10	18471794	0.3827	0.0321	7.66126E-33	rs1623474	T	C	0.3303
10	28233469	0.2527	0.0301	4.64836E-17	rs3802517	A	T	0.4618
10	64564892	-0.3028	0.0309	1.04496E-22	rs2236295	T	G	0.3978
10	96023077	-0.4501	0.0304	1.13789E-49	rs57866767	C	T	0.4322
10	107158054	0.864	0.1199	5.79562E-13	rs117464403	A	G	0.0183
10	115717311	0.5905	0.0782	4.42385E-14	rs60444686	A	G	0.0406
10	124234880	0.236	0.0427	3.16097E-08	rs7093894	A	C	0.1512
10	134459388	0.2975	0.0376	2.52813E-15	rs1133400	G	A	0.214
11	28512458	0.2336	0.0317	1.64816E-13	rs871004	A	G	0.3481
11	48109948	0.2081	0.0325	1.57699E-10	rs2904315	G	A	0.6869
11	99998431	-0.2087	0.038	4.12401E-08	rs67885470	T	C	0.2094
11	100610546	0.655	0.0339	2.54624E-83	rs604723	C	T	0.7244
11	107086143	-0.2603	0.0321	5.71084E-16	rs7926110	G	T	0.3267
11	2114221	0.4404	0.0757	6.07393E-09	rs74048190	C	T	0.0478
11	10679441	-0.1969	0.0318	5.65796E-10	rs1544861	C	T	0.6605
11	45351729	-0.277	0.0411	1.60103E-11	rs11604357	A	C	0.1622
11	47676170	0.4598	0.0301	1.63005E-52	rs7107356	G	A	0.5041

© 2023 Triozzi JL et al. *JAMA Network Open.*

11	65390803	-0.3963	0.0365	1.79391E-27	rs66864335	A	G	0.2211
11	69262916	-0.2162	0.0308	2.1928E-12	rs7395791	A	G	0.4419
11	89224477	-0.308	0.0415	1.13501E-13	rs2289124	A	G	0.1673
11	130469044	0.1754	0.0319	3.74498E-08	rs10750441	T	C	0.6621
11	1888614	0.5764	0.032	2.46718E-72	rs4980379	T	C	0.3719
11	10364963	0.3472	0.0304	2.73905E-30	rs2957688	A	G	0.4707
11	22492454	0.4117	0.0571	5.59887E-13	rs17762	A	G	0.0777
11	27273967	-0.1917	0.0307	4.46601E-10	rs1382472	A	G	0.4041
11	58407740	-0.3296	0.0351	6.55541E-21	rs2276153	G	C	0.243
11	69825414	-0.3967	0.0529	6.4003E-14	rs72931748	G	A	0.0985
11	117074229	0.3193	0.044	3.73766E-13	rs641620	C	T	0.1453
11	117267884	-0.1994	0.0303	4.772E-11	rs573455	G	A	0.539
11	16365282	0.5169	0.0373	1.25893E-43	rs2014408	T	C	0.2087
11	16930289	0.373	0.0442	3.46817E-17	rs177551	A	C	0.1344
11	57448117	0.2622	0.0363	5.08394E-13	rs509564	T	C	0.226
11	67976593	-0.3855	0.0707	4.96604E-08	rs74538877	C	G	0.0557
11	130273230	0.3363	0.0316	1.80302E-26	rs11222084	T	A	0.3621
11	30182068	-0.1936	0.0312	5.77404E-10	rs1340030	C	T	0.3655
11	72088806	0.4122	0.0607	1.10205E-11	rs10501410	A	G	0.0692
12	434755	0.2449	0.0346	1.47911E-12	rs78998485	G	C	0.2557
12	53450097	0.4788	0.0562	1.57906E-17	rs7134440	T	C	0.0822
12	90060586	-0.8446	0.0403	1.24997E-97	rs17249754	A	G	0.1683
12	90349999	-0.1786	0.0299	2.452E-09	rs10777213	A	G	0.5244
12	102837863	0.2233	0.0349	1.525E-10	rs5742643	C	T	0.7513
12	133086888	0.3154	0.0499	2.66397E-10	rs117206641	T	C	0.1108
12	8837407	-0.1835	0.033	2.61999E-08	rs113695818	T	C	0.3032
12	20000315	-0.3571	0.0387	3.01995E-20	rs1010064	C	A	0.1837
12	26457650	0.2643	0.0361	2.44287E-13	rs2129869	T	A	0.2222
12	48210787	0.3427	0.0514	2.67979E-11	rs61917655	T	C	0.1014
12	54441498	-0.3851	0.0332	4.45554E-31	rs7134677	T	C	0.2978
12	79959658	-0.2641	0.0404	6.29651E-11	rs7980644	G	A	0.8334
12	2436837	0.1875	0.0306	9.43604E-10	rs3819532	C	T	0.6087
12	12888438	-0.2642	0.0306	5.87625E-18	rs2024385	A	T	0.424
12	20373541	-0.3962	0.0321	5.52077E-35	rs73075659	G	A	0.3346
12	50573037	-0.3775	0.0309	2.3142E-34	rs12426261	G	A	0.6208
12	67782397	-0.2187	0.0352	5.10399E-10	rs4143175	C	T	0.7591
12	79685226	0.2362	0.0311	2.87276E-14	rs7963801	C	T	0.5779
12	122416254	0.1971	0.0327	1.67699E-09	rs1169078	G	C	0.3121
12	50080878	-0.2785	0.051	4.76903E-08	rs11834380	A	C	0.0968
12	115920472	0.2897	0.0309	6.6115E-21	rs6490019	G	A	0.6204
12	66376091	0.2429	0.0303	1.02494E-15	rs7306710	C	T	0.519

© 2023 Triozzi JL et al. *JAMA Network Open.*

12	111865049	-0.585	0.0306	1.31795E-81	rs7310615	G	C	0.5184
12	115342956	-0.2797	0.0371	4.40453E-14	rs1896326	A	G	0.2291
12	115552437	-0.4368	0.031	3.46737E-45	rs35444	G	A	0.3862
13	73131694	0.1899	0.0312	1.15199E-09	rs17245822	C	A	0.3733
13	115000650	0.4101	0.0423	3.35197E-22	rs7331680	T	G	0.1491
13	56398286	0.2659	0.0418	1.945E-10	rs75961402	A	G	0.1534
13	22294117	0.2709	0.0313	5.09331E-18	rs483071	T	C	0.6248
13	27951090	-0.3208	0.0542	3.23303E-09	rs9507885	T	C	0.0953
13	30146201	-0.3557	0.0353	6.34308E-24	rs9508495	T	C	0.7565
13	41967193	0.2968	0.0406	2.48428E-13	rs4274337	G	A	0.8303
13	47180671	0.2163	0.0362	2.37498E-09	rs7491248	A	G	0.2239
13	51489186	-0.2039	0.0323	2.76802E-10	rs9526707	A	G	0.3216
13	73826901	0.4699	0.0734	1.51199E-10	rs78474310	G	A	0.0448
13	74223828	-0.178	0.0304	4.95496E-09	rs6562778	G	A	0.5411
13	113652369	0.2846	0.05	1.24899E-08	rs9549627	A	G	0.1175
14	23861811	-0.225	0.0312	5.95251E-13	rs365990	G	A	0.3658
14	69260028	0.2317	0.0374	5.626E-10	rs57786342	A	G	0.2059
14	98590629	0.253	0.0309	2.72082E-16	rs7154723	A	G	0.385
14	35871217	0.3061	0.0314	1.71317E-22	rs8904	A	G	0.3678
14	50735947	-0.9587	0.1101	3.08035E-18	rs72683923	C	T	0.0212
14	73422259	0.285	0.05	1.20801E-08	rs3815460	G	C	0.1024
14	39400917	0.189	0.0316	2.30802E-09	rs7493678	T	A	0.3486
14	53420358	0.3002	0.0328	5.25049E-20	rs35413927	G	A	0.3054
14	68032235	-0.2382	0.0428	2.69998E-08	rs12883810	T	C	0.1462
14	75074316	0.1978	0.0303	6.79204E-11	rs11159091	A	G	0.4615
14	100197940	-0.2513	0.0439	1.04701E-08	rs75016974	T	C	0.1423
14	104007555	0.2291	0.0367	4.32305E-10	rs12885878	G	A	0.7663
14	71451265	-0.1755	0.0319	3.59898E-08	rs8003103	A	G	0.3447
14	100133250	0.1967	0.0306	1.34899E-10	rs17562391	T	C	0.4186
15	63406170	-0.2516	0.0353	1.02707E-12	rs2652812	T	C	0.7544
15	75114322	-0.5313	0.0328	4.22377E-59	rs11636952	C	T	0.6859
15	86064327	-0.2513	0.0391	1.23299E-10	rs1994158	G	A	0.1807
15	90023558	-0.2721	0.0443	8.42694E-10	rs17807723	A	G	0.138
15	91429287	0.635	0.0328	2.49E-83	rs4932373	C	A	0.3258
15	95312071	0.2653	0.0325	3.27718E-16	rs12906962	C	T	0.324
15	40314967	0.1764	0.031	1.21199E-08	rs8030856	G	C	0.3953
15	96785017	0.2258	0.0339	2.54273E-11	rs2589218	C	T	0.2703
15	100087596	-0.3196	0.0523	9.71091E-10	rs4606697	A	G	0.1041
15	41442195	0.2762	0.0302	5.45255E-20	rs28866311	G	T	0.4737
15	66931617	0.215	0.0325	3.88776E-11	rs28429256	A	G	0.3342
15	81006712	0.3208	0.0303	3.55222E-26	rs2627313	T	C	0.4454

© 2023 Triozzi JL et al. *JAMA Network Open.*

15	48939888	0.4162	0.0517	7.75711E-16	rs4775769	G	T	0.9055
15	50810621	-0.2422	0.0303	1.41091E-15	rs3098186	T	C	0.5156
16	51704452	0.2015	0.0307	4.9272E-11	rs4784541	C	T	0.5252
16	87984477	-0.2016	0.0322	3.92898E-10	rs6540119	T	A	0.666
16	4932929	0.299	0.0302	4.58987E-23	rs1049212	G	A	0.5693
16	60635748	-0.1967	0.0356	3.19801E-08	rs35098810	C	A	0.2317
16	66781040	0.4824	0.0778	5.63806E-10	rs146550789	C	T	0.0417
16	2065666	0.4278	0.0547	5.01072E-15	rs12596630	T	C	0.0903
16	4138378	-0.3106	0.0481	1.07701E-10	rs2283500	C	A	0.1102
16	70729954	0.5115	0.0686	9.29394E-14	rs62047964	T	C	0.0622
16	74171973	0.192	0.0302	1.94998E-10	rs1012089	G	C	0.5248
16	86436343	0.1851	0.0308	1.82398E-09	rs3950627	A	C	0.531
16	89697625	-0.2261	0.0315	7.1351E-13	rs908951	T	C	0.4378
16	1347717	-0.1943	0.0309	3.26002E-10	rs11641374	A	C	0.5995
16	21088031	-0.2315	0.0302	1.88191E-14	rs7186298	T	C	0.4295
16	75432824	0.3653	0.0307	1.41514E-32	rs4888408	A	G	0.5855
16	81510155	-0.2548	0.0324	3.4261E-15	rs12926550	A	G	0.3156
16	11945778	-0.1824	0.0313	5.99405E-09	rs7198817	A	C	0.6323
16	20392332	-0.4081	0.039	1.12305E-25	rs77924615	A	G	0.1986
16	24811207	-0.2138	0.0331	1.068E-10	rs8044992	C	T	0.2877
16	50550137	-0.3225	0.0425	3.2337E-14	rs34941092	A	G	0.1498
16	86170044	-0.1665	0.0302	3.41099E-08	rs8054587	C	T	0.4728
17	7815712	-0.4688	0.0559	4.89892E-17	rs79930761	T	C	0.0872
17	76799898	-0.2242	0.0302	1.03395E-13	rs9302885	G	A	0.5548
17	1371473	0.2101	0.0325	1.02601E-10	rs8079811	G	C	0.6521
17	18185510	0.2174	0.0305	9.65606E-13	rs4925159	A	G	0.4246
17	30777924	-0.2011	0.0316	1.865E-10	rs9899540	T	A	0.6001
17	43155914	-0.4	0.0315	6.24022E-37	rs7213273	A	G	0.655
17	45013271	0.6903	0.0433	2.47913E-57	rs17608766	C	T	0.1445
17	61090958	-0.2363	0.0377	3.78504E-10	rs62076622	G	A	0.1987
17	7455536	-0.2881	0.0318	1.27909E-19	rs4511593	T	C	0.6528
17	30032420	0.2098	0.0356	3.88597E-09	rs1551355	T	C	0.2334
17	47518378	0.2645	0.0319	1.18905E-16	rs9897429	A	G	0.52
17	59475642	0.4138	0.0346	6.5013E-33	rs1000423	T	C	0.7316
17	60767135	0.2178	0.031	2.01094E-12	rs56288724	G	A	0.4169
17	46123932	-0.3748	0.0445	3.68893E-17	rs3764400	C	T	0.1365
17	79365861	-0.2033	0.0333	1.086E-09	rs11655604	T	C	0.3579
17	2001604	0.3626	0.0509	1.0539E-12	rs2760748	A	T	0.0981
17	7171356	0.3249	0.0307	3.80277E-26	rs113086489	T	C	0.5525
17	19922364	0.1779	0.0304	4.61201E-09	rs7211535	G	A	0.5236
17	62381714	0.2982	0.0312	1.24796E-21	rs6504213	C	T	0.5818

© 2023 Triozzi JL et al. *JAMA Network Open.*

17	75316880	-0.3119	0.0315	4.72716E-23	rs1436138	G	A	0.3633
18	777282	-0.3531	0.0393	2.46888E-19	rs34413141	A	T	0.1822
18	54578482	-0.2607	0.0317	1.90722E-16	rs10048404	T	C	0.3701
18	42179819	0.3603	0.034	2.78099E-26	rs56407827	T	C	0.2687
18	43097750	0.3431	0.0388	8.51138E-19	rs7236548	A	C	0.1848
18	51842682	-0.1909	0.0334	1.15199E-08	rs665445	A	C	0.2794
18	22676071	-0.1884	0.0345	4.689E-08	rs62082230	A	T	0.2773
18	42596789	0.2856	0.0328	3.22478E-18	rs11874246	T	C	0.2963
18	48132646	-0.2189	0.0357	8.57294E-10	rs1437649	A	G	0.2345
18	73034151	-0.2141	0.0301	1.11995E-12	rs10460108	G	A	0.5199
18	24546824	0.2031	0.0306	3.27416E-11	rs1154214	G	T	0.6037
19	2165383	0.7807	0.078	1.35613E-23	rs141958336	A	G	0.043
19	18455444	-0.22	0.0345	1.793E-10	rs34518929	A	G	0.2623
19	30350005	-0.3395	0.0619	4.14295E-08	rs60138042	G	C	0.0645
19	32591878	-0.228	0.0326	2.65522E-12	rs1433121	T	C	0.6906
19	46180184	0.2212	0.0381	6.31902E-09	rs11672660	T	C	0.1996
19	5006598	-0.2315	0.032	4.40656E-13	rs12610654	G	A	0.3436
19	7257990	-0.845	0.049	1.228E-66	rs12978472	G	C	0.1241
19	11508177	0.3708	0.0505	2.16521E-13	rs2291516	A	G	0.103
19	45298461	-0.4921	0.0887	2.85601E-08	rs10420519	T	G	0.0347
19	49207554	0.228	0.0304	6.7655E-14	rs571689	T	C	0.5196
19	10350649	-0.2334	0.0398	4.58701E-09	rs8113613	T	C	0.1813
19	22111366	0.2012	0.0334	1.78698E-09	rs1848994	A	G	0.2828
19	31867447	0.2733	0.0308	6.34016E-19	rs28572357	C	A	0.3977
19	45766729	0.2306	0.0345	2.44118E-11	rs7255933	A	G	0.2574
19	49605705	-0.3554	0.0426	7.23436E-17	rs73046792	A	G	0.1588
20	10967214	0.4274	0.03	5.38146E-46	rs6108787	G	T	0.4704
20	51788718	0.1876	0.0324	7.36902E-09	rs2801008	G	T	0.3183
20	6327810	0.2094	0.0306	8.22811E-12	rs6054139	A	G	0.606
20	10693337	-0.3011	0.0302	1.76685E-23	rs2423514	G	A	0.4589
20	11168669	-0.1849	0.0304	1.19501E-09	rs6078093	A	G	0.428
20	17883531	0.1761	0.0301	4.84295E-09	rs8125763	A	C	0.4717
20	30139886	-0.2832	0.0417	1.14393E-11	rs6058088	G	T	0.1561
20	40266681	-0.2712	0.033	1.88408E-16	rs6029756	A	G	0.3225
20	47241618	-0.168	0.0303	2.86999E-08	rs2598	G	A	0.467
20	47410231	-0.3854	0.0425	1.28588E-19	rs6090907	A	G	0.147
20	57463472	0.185	0.0316	4.59399E-09	rs6026578	G	C	0.6269
20	31214944	0.3179	0.0428	1.07696E-13	rs79384779	T	C	0.1512
20	57742388	0.7131	0.0461	6.99842E-54	rs6026744	T	A	0.1229
20	19007099	-0.3613	0.0525	5.64547E-12	rs17812022	T	C	0.0958
20	42795152	0.2617	0.0304	7.04855E-18	rs6031431	G	A	0.4624

© 2023 Triozzi JL et al. *JAMA Network Open.*

20	62446351	-0.3294	0.0363	1.18413E-19	rs6062324	A	G	0.2364
21	33814378	0.2177	0.0338	1.217E-10	rs2833834	A	C	0.2765
21	44760603	0.3498	0.0341	1.024E-24	rs12627651	A	G	0.2872
21	16317933	0.1851	0.0309	2.146E-09	rs2776037	C	T	0.5849
21	16556367	0.2443	0.0326	6.68652E-14	rs1882961	T	C	0.3087
21	44838330	-0.8819	0.1244	1.3499E-12	rs34487963	A	C	0.0185
21	44966069	0.1876	0.0304	6.62796E-10	rs7278003	C	T	0.5622
22	30588910	0.5577	0.1004	2.77402E-08	rs112854918	G	C	0.0255
22	19976406	0.2552	0.0332	1.45111E-14	rs2238787	A	G	0.292
22	50727921	-0.2066	0.0327	2.52802E-10	rs28578714	C	T	0.3938
22	29453193	-0.2292	0.0303	3.81417E-14	rs12321	C	G	0.4328
22	32001037	0.1676	0.03	2.19498E-08	rs8142376	T	C	0.491
22	50228044	-0.3252	0.0562	7.39095E-09	rs148140538	T	C	0.0808

Abbreviation: chr, chromosome; se, standard error; ea, effect allele; nea, non-effect allele; eaf, effect allele frequency.

© 2023 Triozzi JL et al. *JAMA Network Open.*

eTable 5. F-statistics for instrumental variables.

Exp	nsnp	min F-statistic	max F-statistic	median F-statistic
TZ	5	15.76403	28.01639	20.10598
BB	21	14.68085	177.5439	31.64521
SBP	461	29.7205	627.5475	50.25716

Abbreviation: Exp, exposure; nsnp, number of single nucleotide polymorphisms; min, minimum; max, maximum.

© 2023 Triozzi JL et al. *JAMA Network Open.*

eTable 6. Harmonization of genetic proxies of thiazide diuretics with kidney stone risk in the Million Veteran Program, UK Biobank, and FinnGen study.

SNP	MVP beta	MVP se	MVP pval	UKB beta	UKB se	UKB pval	FinnGen beta	FinnGen se	FinnGen pval
rs13306677	-0.0363	0.013036	0.001491	-0.0325	0.03288	0.6121	-0.0487	0.0318	0.1257
rs35797045	-0.0564	0.019031	0.001357	-0.00511	0.04595	0.9605	-0.0429	0.0398	0.2818
rs7500207	0.0236	0.008367	0.003955	0.005941	0.02163	0.8995	0.0253	0.0214	0.2363
rs9925265	0.028	0.007653	0.00018	0.03498	0.01967	0.3253	0.0402	0.0205	0.04998

Abbreviation: MVP, Million Veteran Program; UKB, UK Biobank; FinnGen, FinnGen Study; SNP, single nucleotide polymorphism; se, standard error.

© 2023 Triozzi JL et al. *JAMA Network Open.*

eTable 7. Harmonization of genetic proxies of beta blockers with kidney stone risk in the Million Veteran Program, UK Biobank, and FinnGen study.

SNP	MVP beta	MVP se	MVP pval	UKB beta	UKB se	UKB pval	FinnGen beta	FinnGen se	FinnGen pval
rs10787510	0.001	0.009082	0.9137	0.01027	0.01989	0.8043	0.0011	0.0208	0.9589
rs10885595	0.006	0.00875	0.4976	-0.00371	0.01952	0.9314	0.024	0.0207	0.2452
rs11196597	-0.005	0.013699	0.6872	0.00887	0.02894	0.8873	0.0201	0.0309	0.5155
rs11196621	-0.0169	0.008929	0.04424	0.01749	0.0223	0.695	-0.0053	0.0241	0.8267
rs11817866	-0.003	0.008673	0.7352	-0.01589	0.01971	0.6861	-0.009	0.0205	0.6595
rs139275657	-0.0583	0.038801	0.1315	-0.05347	0.06943	0.7009	-0.0281	0.1085	0.7958
rs17091184	-0.0109	0.022577	0.6222	-0.0016	0.04777	0.9883	-0.0272	0.0434	0.531401
rs17091398	0.0154	0.012398	0.2108	0.02753	0.02896	0.6274	0.0265	0.0315	0.3998
rs17875473	-0.008	0.015281	0.5885	0.03587	0.03527	0.6006	-0.0148	0.0326	0.6505
rs2429511	-0.0139	0.007628	0.04956	0.02152	0.01945	0.565	-0.0398	0.0204	0.05146
rs2782980	-0.0208	0.00875	0.007284	0.03109	0.02166	0.4403	-0.0239	0.0215	0.266
rs4256930	-0.0198	0.007755	0.01341	-0.03222	0.02026	0.386	-0.0161	0.0204	0.4314
rs460718	-0.003	0.009107	0.7618	0.01843	0.02077	0.653	-0.021	0.025	0.4
rs56012176	-0.0169	0.012781	0.182	0.02047	0.0289	0.7262	0.0234	0.035	0.5037
rs68122733	0.0155	0.01148	0.1764	-0.01736	0.02572	0.7399	0.017	0.0242	0.4838
rs7086922	0.0042	0.020179	0.8352	0.01323	0.04797	0.8991	-4.00E-04	0.054	0.9941
rs740746	-0.009	0.009388	0.327	0.02398	0.02205	0.5725	-0.0353	0.0234	0.1323
rs78793615	0.0335	0.030587	0.2734	-0.00571	0.06958	0.9711	0.0928	0.0828	0.2625
rs7894582	0.0079	0.020102	0.6947	0.06839	0.04769	0.4407	0.0397	0.0376	0.2919
rs79850079	0.021	0.02625	0.4215	0.01862	0.05637	0.878	0.0068	0.0698	0.9222
rs855715	0.0159	0.015638	0.3064	-0.04602	0.03023	0.4095	0.0392	0.037	0.289

Abbreviation: MVP, Million Veteran Program; UKB, UK Biobank; FinnGen, FinnGen Study; SNP, single nucleotide polymorphism; se, standard error.

© 2023 Triozzi JL et al. *JAMA Network Open.*

eTable 8. Harmonization of genetic proxies of systolic blood pressure with kidney stone risk in the Million Veteran Program, UK Biobank, and FinnGen study.

SNP	MVP beta	MVP se	MVP pval	UKB beta	UKB se	UKB pval	FinnGen beta	FinnGen se	FinnGen pval
rs1000423	0.0735	0.008852	4.61E-20	0.04467	0.022	0.2531	0.0799	0.0228	0.000462
rs10008637	-0.005	0.008444	0.5275	0.04004	0.01944	0.2456	0.0336	0.0204	0.1004
rs10028284	0.0158	0.011097	0.1534	0.01151	0.02558	0.8309	-0.0105	0.0235	0.655799
rs10045307	-0.0188	0.010179	0.07068	0.003086	0.02322	0.9526	-0.0258	0.0235	0.2714
rs10048404	0.0041	0.010102	0.6825	-0.01004	0.02009	0.8111	0.0204	0.0221	0.3543
rs1006545	-0.0325	0.011837	0.004855	-0.02777	0.03067	0.6457	0.0059	0.0423	0.889
rs10069690	-0.004	0.009566	0.6859	0.0141	0.02217	0.7557	0.0369	0.0224	0.098569
rs1010064	0.0075	0.011301	0.5047	0.02889	0.02506	0.5467	0.0111	0.0311	0.7199
rs1012089	-0.009	0.008673	0.2736	0.02436	0.01949	0.5092	0.0109	0.0205	0.5942
rs10188003	-0.006	0.008699	0.5229	-0.03537	0.01988	0.325	-0.0087	0.0207	0.673899
rs10207726	-0.007	0.009388	0.4422	0.03018	0.02132	0.4474	-0.0219	0.0206	0.2874
rs10224210	-0.0554	0.008673	8.61E-12	-0.09482	0.02158	0.0071	-0.0314	0.0243	0.1966
rs10282122	0.0202	0.008214	0.003786	0.0157	0.02059	0.7041	0.0267	0.0233	0.2527
rs10420519	0.0111	0.02273	0.6253	0.09019	0.05363	0.3559	0.0352	0.0681	0.6055
rs1044822	0.0163	0.012296	0.1811	-0.01114	0.02701	0.8459	0.0332	0.0322	0.3017
rs10460108	0.015	0.008622	0.07914	0.003631	0.01952	0.933	0.0113	0.0204	0.5807
rs1049212	0.0249	0.0075	0.00057	0.04383	0.01959	0.2025	0.0127	0.0208	0.5407
rs10501410	-0.0247	0.0175	0.1567	0.009019	0.03906	0.9162	-0.0836	0.0329	0.01115
rs1052501	0.018	0.010842	0.1009	0.01525	0.02648	0.282339	-0.0851	0.0256	0.000892
rs10749572	0.0026	0.008469	0.756	0.03565	0.01948	0.3094	0.0096	0.0207	0.6438
rs10750441	0.0071	0.008724	0.4133	-0.01038	0.02063	0.8096	-0.0151	0.0211	0.473301
rs10776752	0.0372	0.014439	0.003824	-0.03808	0.0372	0.598	0.0354	0.0274	0.1954
rs10777213	0.0092	0.008393	0.2754	-0.02189	0.01942	0.5567	0.0166	0.0205	0.4171
rs10779795	-0.006	0.008929	0.4838	-0.06213	0.02057	0.0744	-0.0166	0.0226	0.4626
rs10782230	0.0047	0.009541	0.6269	-0.03089	0.01941	0.3857	-0.022	0.0204	0.281

© 2023 Triozzi JL et al. *JAMA Network Open.*

rs10804330	-0.005	0.008673	0.5837	-0.05605	0.01976	0.0962	-0.0121	0.0206	0.5573
rs10914124	-0.005	0.008954	0.5494	0.01497	0.01993	0.7087	0.0362	0.0207	0.08085
rs10941043	0.0045	0.009362	0.6238	0.01227	0.02133	0.7804	0.0414	0.0225	0.06558
rs10980408	0.0226	0.023495	0.3356	-0.04672	0.0527	0.6534	-0.1347	0.0904	0.1362
rs11097909	0.0057	0.011633	0.6286	0.02928	0.0273	0.5785	-0.0409	0.033	0.2157
rs11120093	-0.0129	0.00852	0.1331	0.01857	0.01974	0.6313	-0.0265	0.0208	0.2029
rs11145807	0.0146	0.008903	0.09858	-0.00735	0.01988	0.8626	-0.0222	0.0209	0.2879
rs11159091	0.0179	0.00773	0.01476	-0.00781	0.01952	0.8506	0.0285	0.0206	0.1677
rs111866816	-0.0257	0.018061	0.1552	-0.03104	0.03777	0.6798	-0.0194	0.0494	0.6951
rs11191580	-0.004	0.014643	0.7911	-0.00321	0.03629	0.9687	0.0331	0.0366	0.3661
rs11210029	-0.0208	0.007985	0.01011	0.009417	0.02023	0.8247	0.007	0.0209	0.7361
rs11222084	0.001	0.009082	0.9142	0.03948	0.02008	0.2706	-0.011	0.0217	0.6125
rs11241313	0.0029	0.009082	0.7504	0.0214	0.021	0.5998	-0.0156	0.0227	0.4909
rs112509803	-0.005	0.013622	0.7253	0.001422	0.03012	0.9835	0.0208	0.0332	0.531401
rs11252324	-0.01	0.015867	0.5305	0.07545	0.03692	0.2498	0.1002	0.0466	0.03145
rs112854918	0.002	0.02824	0.9443	-0.0318	0.06524	0.816	-0.074	0.0519	0.1539
rs113086489	-0.0109	0.008903	0.2092	-0.02489	0.01961	0.5018	-0.0109	0.0208	0.600101
rs1133400	-0.0354	0.009107	1.64E-05	-0.04988	0.02334	0.2263	-0.0603	0.0271	0.02611
rs115262049	3.00E-04	0.015714	0.9848	-0.04217	0.03426	0.5164	0.047	0.0388	0.2255
rs1154214	0.0074	0.008852	0.3985	0.002587	0.01984	0.9535	0.0284	0.0207	0.1698
rs11585169	0.0086	0.008495	0.3119	0.04453	0.01968	0.1967	0.0283	0.0207	0.1711
rs11592107	0.0161	0.00824	0.04045	0.04048	0.02096	0.2803	4.00E-04	0.0224	0.986
rs11604357	-0.003	0.011709	0.8263	3.27E-05	0.02646	0.9996	-0.0364	0.0308	0.2369
rs11636952	0.0131	0.009107	0.1475	0.0268	0.02113	0.5021	0.0217	0.021	0.3006
rs11641374	0.0075	0.008903	0.3948	0.03631	0.01984	0.3097	0.0129	0.0217	0.551899
rs11655604	-0.0149	0.007985	0.03823	-0.01327	0.02024	0.7477	-0.0119	0.0213	0.5771
rs11672660	-0.0853	0.009388	3.77E-21	-0.08055	0.02457	0.0507	-0.0605	0.0233	0.009418
rs1169078	-0.0208	0.007985	0.01408	-0.00085	0.02096	0.9858	0	0.0213	0.9987
rs11694601	-0.0119	0.009286	0.2179	0.008609	0.01998	0.8385	-0.0055	0.0207	0.791799
rs117206641	-0.0296	0.011862	0.004702	-0.05721	0.03142	0.3122	-0.0339	0.0366	0.3553

© 2023 Triozzi JL et al. *JAMA Network Open.*

rs117464403	0.0141	0.033469	0.6731	-0.05767	0.07087	0.6831	0.0299	0.0667	0.654201
rs11834380	0.0098	0.014107	0.4866	-0.06073	0.03315	0.3091	-0.0146	0.0308	0.6359
rs11874246	0.0241	0.008444	0.002563	0.02054	0.02129	0.6215	-0.0178	0.0213	0.403
rs11925504	-0.001	0.008724	0.9167	0.008344	0.01964	0.8409	-0.0195	0.0205	0.3412
rs11960210	-0.0198	0.007755	0.003822	0.0164	0.02008	0.682	-0.0019	0.0215	0.9292
rs11977526	0.0147	0.008495	0.08103	-0.02232	0.01981	0.5569	-0.0138	0.0218	0.5287
rs1199330	-0.008	0.013138	0.5604	-0.05311	0.03032	0.3335	-0.0137	0.0325	0.674
rs12042924	-0.002	0.008597	0.8135	-0.00491	0.0195	0.9082	-0.0206	0.0205	0.3143
rs12063372	-0.008	0.009439	0.3873	-0.00308	0.02015	0.9454	-0.0074	0.021	0.723801
rs1209384	-0.006	0.009541	0.5259	0.000305	0.01988	0.9947	-0.0024	0.0215	0.9107
rs12136922	0.0013	0.008367	0.8776	-0.00478	0.01944	0.9105	0.0189	0.0205	0.3585
rs12153395	0.002	0.014643	0.8897	0.06339	0.03048	0.2405	0.047	0.03	0.1174
rs12255372	2.00E-04	0.009184	0.9791	0.009206	0.02139	0.8387	-0.0416	0.0264	0.1155
rs12258967	0.0036	0.009872	0.7109	0.01613	0.02123	0.7051	0.0314	0.0246	0.2031
rs12264186	-0.001	0.010842	0.9555	0.03942	0.02486	0.3877	-0.0042	0.03	0.8888
rs12321	-0.0198	0.007755	0.006231	-0.04225	0.01955	0.2202	-0.0054	0.0209	0.796
rs12426261	-0.002	0.008622	0.8306	0.01308	0.02008	0.7494	0.0044	0.0211	0.8338
rs12464602	0.0028	0.009107	0.7626	-0.03895	0.02021	0.2814	0.0062	0.0208	0.764999
rs12509595	0.0049	0.009209	0.5937	-0.0022	0.02135	0.9635	0.0429	0.022	0.051171
rs12511987	0.0122	0.011352	0.2829	0.004325	0.02553	0.9392	-0.0501	0.0266	0.05938
rs12528975	-0.0119	0.019668	0.5407	0.07995	0.04493	0.325	-0.0239	0.0546	0.661899
rs12596630	-0.005	0.01523	0.7621	0.01645	0.03416	0.8182	-0.0398	0.0447	0.3736
rs12610654	-0.0109	0.009056	0.234	-0.00392	0.0205	0.9311	-0.0067	0.0205	0.744
rs12627651	-0.0208	0.009235	0.01838	-0.01804	0.02155	0.6735	0.0055	0.022	0.8025
rs12637573	-0.0218	0.007474	0.002987	-0.04113	0.01948	0.2325	-0.0443	0.0204	0.02978
rs12643599	0.0203	0.007781	0.004219	-0.0053	0.02018	0.9042	-0.0129	0.0217	0.5535
rs12656497	0.0151	0.008673	0.07748	0.006339	0.01978	0.8819	-0.0171	0.0206	0.4077
rs12657950	0.0291	0.016862	0.08217	0.01439	0.0387	0.8618	-0.0298	0.0288	0.3014
rs12661036	0.0236	0.009949	0.01332	0.006207	0.02369	0.9043	0.0015	0.0226	0.9482
rs12668436	0.0146	0.009974	0.1407	-0.03857	0.02247	0.3447	0.0169	0.0233	0.4679

© 2023 Triozzi JL et al. *JAMA Network Open.*

rs12693982	0.0091	0.008673	0.2869	-0.02266	0.01979	0.5499	-0.0114	0.0204	0.578
rs12694277	0.0098	0.009541	0.2993	-0.00658	0.0217	0.8884	0.0051	0.0225	0.8197
rs12731646	-0.0198	0.00801	0.01182	-0.01074	0.01976	0.7933	0.0047	0.0206	0.8198
rs1275985	-0.01	0.008571	0.2512	-0.04587	0.01999	0.1898	0.0157	0.0205	0.4441
rs12883810	-0.0109	0.012015	0.3641	0.01636	0.02757	0.7731	-0.012	0.0282	0.671399
rs12885878	-0.0169	0.010918	0.122	0.01727	0.02378	0.7189	-0.0169	0.0227	0.4561
rs12906962	0.0049	0.009184	0.5929	-0.01294	0.02095	0.7632	-0.0086	0.0209	0.6817
rs1290784	1.00E-04	0.008571	0.99	0.0314	0.01954	0.3805	0.0064	0.0207	0.758501
rs1290933	0.0048	0.009082	0.5977	0.001047	0.02092	0.9825	-0.053	0.0231	0.02172
rs12926550	-0.0129	0.009439	0.1696	-0.00608	0.02093	0.8935	0.0228	0.023	0.3222
rs1293969	-0.0188	0.009643	0.05689	-0.00451	0.02253	0.9277	-0.038	0.0223	0.088379
rs12978472	-0.0257	0.01324	0.05265	-0.0035	0.02908	0.9572	0.0805	0.0411	0.0503
rs13016772	0.0195	0.010026	0.05382	0.02561	0.02283	0.559	-0.0045	0.0259	0.8621
rs13091418	0.009	0.008724	0.3062	0.003841	0.02044	0.9323	-0.008	0.022	0.7149
rs13107261	-0.0139	0.008801	0.1064	-0.00441	0.02011	0.9205	-0.0076	0.0214	0.724499
rs13107325	-0.007	0.015128	0.6198	-0.02142	0.03682	0.280368	0.0656	0.0842	0.4356
rs13204703	0.0188	0.00875	0.01848	0.03108	0.02257	0.4614	0.0255	0.0238	0.2844
rs13253358	0.0063	0.009311	0.493	0.009544	0.02116	0.8304	0.0408	0.0227	0.07201
rs1332813	-0.003	0.009107	0.7324	-0.04843	0.02038	0.1726	0.0025	0.022	0.9079
rs13358657	0.0042	0.012321	0.7325	0.02861	0.02856	0.6068	0.0604	0.0312	0.05291
rs1340030	0.009	0.00875	0.3043	0.01568	0.02013	0.6973	-9.00E-04	0.0207	0.9662
rs13412750	-0.006	0.01	0.5534	-0.03683	0.02161	0.3485	-0.0375	0.027	0.1636
rs13420463	-0.0218	0.00898	0.008357	-0.02517	0.02306	0.571	-0.0042	0.0247	0.8663
rs1375564	0.0026	0.008852	0.7682	-0.01503	0.02034	0.7136	-0.0081	0.0227	0.7206
rs1382472	-0.0218	0.00773	0.002403	-0.03057	0.01971	0.3993	-0.0131	0.0218	0.549301
rs139354822	-0.0149	0.025714	0.5716	-0.0293	0.06066	0.8177	0.0546	0.0497	0.272
rs1408945	0.0096	0.008469	0.2572	0.02201	0.01961	0.5586	-0.0171	0.0207	0.4075
rs1410222	0.0074	0.011276	0.5038	-0.00746	0.02493	0.8901	0.0273	0.027	0.312
rs141958336	-0.0227	0.023316	0.3272	-0.03599	0.04985	0.7206	-0.0307	0.0355	0.3867
rs1422279	-0.0159	0.008546	0.06559	-0.02024	0.02003	0.6032	-0.0015	0.0208	0.9423

© 2023 Triozzi JL et al. *JAMA Network Open.*

rs1433121	0.0276	0.008367	0.000923	0.01301	0.02116	0.7644	0.0132	0.0211	0.533301
rs1436138	-0.008	0.008852	0.3537	-0.03158	0.02031	0.398	-0.0202	0.0213	0.3447
rs1437649	-0.0198	0.010536	0.05207	0.0226	0.02314	0.6169	-0.026	0.0241	0.2799
rs146550789	-0.0139	0.022781	0.5474	-0.002	0.04917	0.9858	0.0179	0.0428	0.6758
rs148140538	0.0273	0.015332	0.07313	-0.01376	0.03547	0.8556	0.0718	0.0466	0.1237
rs148401029	5.00E-04	0.02324	0.9841	-0.04303	0.05241	0.6802	-0.0855	0.0644	0.1843
rs1493132	0.007	0.008776	0.4284	0.02268	0.02047	0.5642	-0.0367	0.0209	0.07856
rs1544861	0.0277	0.008138	0.000622	0.05696	0.0206	0.106	-0.0093	0.0211	0.6598
rs1551355	-0.0169	0.009898	0.08812	-0.00143	0.02304	0.9782	0.0012	0.0233	0.9587
rs1565440	-0.008	0.008827	0.3472	-0.0088	0.02008	0.8357	-0.0226	0.0211	0.2854
rs1575290	-0.009	0.008367	0.2642	0.008975	0.01942	0.826	0.0015	0.0205	0.9406
rs1623474	1.00E-04	0.009082	0.9874	-0.01548	0.02069	0.7099	-0.0275	0.0219	0.2085
rs1630736	0.0058	0.00852	0.4903	0.007992	0.01956	0.8473	0.006	0.0206	0.769699
rs1664781	0.0051	0.009158	0.571	-0.00061	0.02104	0.9899	0.0579	0.0217	0.007522
rs17010957	0.0079	0.012245	0.5216	0.03633	0.0274	0.4803	0.0126	0.0284	0.6575
rs17035181	-0.002	0.012194	0.8922	-0.07236	0.02762	0.1281	-0.0212	0.029	0.466
rs17080102	0.019	0.016709	0.2527	-0.02911	0.03774	0.7005	0.0165	0.0383	0.6659
rs17245822	0.0075	0.009413	0.4268	0.04572	0.02023	0.1971	0.0184	0.0209	0.3775
rs17249754	0.0159	0.011097	0.1497	0.02296	0.02579	0.6518	0.0634	0.0385	0.099431
rs17257081	-0.0208	0.00949	0.01884	-0.04621	0.02585	0.3224	0.0482	0.0245	0.04908
rs17562391	-0.008	0.008495	0.3407	-0.00069	0.01967	0.9877	0.0437	0.0206	0.03378
rs17608766	-0.01	0.012168	0.4284	-0.02935	0.02731	0.5776	-0.003	0.027	0.9102
rs177551	0.0056	0.012398	0.648	-0.01422	0.02844	0.8109	-0.043	0.0329	0.191
rs17760259	-0.002	0.008648	0.8481	-0.01605	0.01965	0.6819	0.0114	0.0205	0.5774
rs17762	-0.0383	0.01523	0.01662	0.006756	0.03805	0.9362	0.033	0.033	0.3173
rs17807723	0.0316	0.010638	0.001209	0.1029	0.02851	0.0452	0.0087	0.0261	0.7395
rs17812022	0.0093	0.017474	0.5948	-0.01936	0.03304	0.7761	-0.0296	0.0328	0.366
rs1786345	0.0052	0.008648	0.547	-0.00693	0.01963	0.8693	0.0194	0.0206	0.3474
rs1814951	-0.004	0.013061	0.7635	-0.03936	0.03043	0.4925	-0.0279	0.026	0.2823
rs1821002	-0.007	0.008444	0.3997	-0.00206	0.01979	0.9631	0.0384	0.0281	0.1713

© 2023 Triozzi JL et al. *JAMA Network Open.*

rs1848994	-0.0159	0.008724	0.03754	-0.00395	0.0216	0.9341	-0.004	0.0222	0.8574
rs1870735	-0.008	0.008724	0.3847	-0.04342	0.01967	0.2091	-0.0046	0.0206	0.8233
rs1871190	-0.0139	0.009337	0.128	-0.02766	0.02079	0.4787	-0.0094	0.0208	0.651799
rs1882212	0.0357	0.009133	4.44E-05	0.02958	0.02321	0.101252	0.003	0.0282	0.9149
rs1882961	-0.0178	0.00852	0.03555	-0.04327	0.02114	0.2488	0.0216	0.021	0.304
rs1889785	0.0315	0.007628	2.93E-05	0.02461	0.0195	0.5044	0.0065	0.0207	0.7526
rs189267552	0.0261	0.043571	0.5494	-0.1127	0.08756	0.495	0.1321	0.0648	0.04152
rs1896326	-0.007	0.011505	0.5456	0.01991	0.02348	0.6692	-0.0208	0.0262	0.4284
rs1906672	-0.0257	0.009184	0.000963	-0.04149	0.02282	0.3132	-0.0233	0.0266	0.3815
rs1957563	-0.0276	0.008444	0.000573	0.01353	0.02185	0.7626	-0.021	0.0243	0.389
rs1984195	7.00E-04	0.008597	0.9311	-0.02555	0.01942	0.4841	-0.0167	0.0204	0.4137
rs1994158	-0.0119	0.010918	0.2706	-0.05219	0.02517	0.242	-0.0433	0.0245	0.076611
rs2014408	-0.003	0.010995	0.7735	-0.01997	0.02376	0.6722	0.0146	0.0283	0.6058
rs2024385	-0.0178	0.009133	0.05572	-0.01871	0.01966	0.6269	-0.0203	0.0211	0.3361
rs2111557	-0.005	0.008622	0.5647	-0.00458	0.01949	0.9146	0.025	0.0204	0.2211
rs2126474	0.0011	0.008699	0.8973	-0.00244	0.01969	0.9559	0.0194	0.0212	0.3606
rs2129869	0.0467	0.009362	1.17E-08	0.0344	0.02346	0.429	0.0122	0.023	0.5953
rs2161967	-0.0218	0.007985	0.003272	-0.03583	0.01969	0.3129	-0.0339	0.0205	0.097911
rs2177843	-0.0315	0.010612	0.005103	-0.07693	0.02762	0.1032	-0.0246	0.0283	0.386
rs2236295	0.0146	0.008903	0.09869	0.01092	0.01984	0.7906	0.0108	0.0212	0.6101
rs2238787	0.0179	0.008444	0.01377	0.03059	0.02146	0.4437	0.0323	0.0211	0.126
rs2249105	-0.0159	0.008699	0.06027	-0.03234	0.02014	0.3809	-0.0453	0.0217	0.03668
rs2276153	4.00E-04	0.010332	0.9724	-0.03589	0.0228	0.3916	0.0036	0.0233	0.8769
rs2283500	-0.0247	0.013316	0.03512	-0.04899	0.03174	0.4021	0.0023	0.0336	0.946
rs2289124	-0.0129	0.012296	0.2808	-0.00115	0.02645	0.9847	0.0207	0.0285	0.4675
rs2291434	-0.0266	0.007449	0.000148	-0.04463	0.01947	0.1901	-0.0018	0.0206	0.9308
rs2291516	-0.002	0.013648	0.8879	-0.02111	0.03269	0.7401	-0.0439	0.0313	0.1609
rs2353940	2.00E-04	0.010816	0.9857	-0.00795	0.02233	0.8681	-0.0106	0.024	0.6598
rs2354862	0.0111	0.00875	0.2077	-0.01035	0.02024	0.8063	-0.0069	0.0239	0.7723
rs2384063	-0.008	0.010026	0.4289	0.0113	0.02283	0.8129	0.0256	0.0237	0.2803

© 2023 Triozzi JL et al. *JAMA Network Open.*

rs2392929	0.0103	0.010332	0.3185	-0.01448	0.02431	0.7721	0.023	0.0224	0.3038
rs2423514	0.0228	0.007883	0.002892	0.005863	0.01944	0.8891	0.0297	0.0208	0.1521
rs246973	-0.0169	0.009005	0.04818	-0.0358	0.02164	0.3646	0.057	0.0223	0.01064
rs2470004	0.0041	0.010816	0.7069	-0.02376	0.02491	0.6261	-0.0511	0.0312	0.1013
rs2493134	-0.0109	0.008418	0.1852	-0.01199	0.0198	0.7681	-0.0159	0.0206	0.4397
rs2493296	-0.008	0.011684	0.5078	0.02461	0.0283	0.6603	0.0157	0.0327	0.630901
rs2498323	-0.007	0.013954	0.6188	-0.03197	0.03235	0.6139	-0.0483	0.0406	0.2347
rs2580350	0.0315	0.00773	5.35E-06	0.02058	0.01967	0.5888	0.0173	0.0207	0.4036
rs2589218	0.0077	0.00949	0.4127	-0.01389	0.02195	0.7572	0.0278	0.0244	0.2558
rs2598	0.0168	0.008648	0.05339	-0.01075	0.01954	0.7906	0.0087	0.0207	0.673101
rs2608029	0.005	0.009184	0.5897	0.01047	0.02062	0.8078	7.00E-04	0.022	0.9739
rs2610990	0.0041	0.009719	0.6723	-0.0141	0.02208	0.7547	0.0025	0.0218	0.9093
rs2627313	0.0018	0.00852	0.8296	0.05593	0.01955	0.093	-0.0133	0.0208	0.5219
rs262986	-0.004	0.008699	0.6717	0.004968	0.01956	0.9074	0.0117	0.0211	0.5777
rs263532	-0.01	0.008597	0.2578	0.000515	0.01967	0.9909	-0.033	0.0205	0.1073
rs2643826	0.0206	0.007806	0.00961	0.02147	0.01954	0.568	0.0274	0.0208	0.188
rs2652812	-0.0139	0.010051	0.1755	-0.00619	0.02308	0.902	-0.0048	0.0224	0.8313
rs268263	-0.01	0.009821	0.2964	-0.01271	0.02267	0.7864	-0.0082	0.0251	0.744501
rs2689690	3.00E-04	0.008852	0.9738	0.03967	0.02012	0.269	0.0175	0.0229	0.4445
rs2724377	0.0138	0.008163	0.09558	-0.01028	0.01943	0.7992	-0.0143	0.0204	0.482
rs2744139	0.0043	0.01023	0.6711	-0.00622	0.02286	0.9004	0.0169	0.0246	0.4925
rs2745599	0.019	0.007602	0.008536	0.03335	0.0196	0.3496	0.0247	0.021	0.2395
rs2760748	-0.0188	0.014541	0.1875	-0.02847	0.03275	0.6604	-0.0089	0.0344	0.7949
rs2776037	-0.001	0.008367	0.8608	-0.03961	0.01977	0.2605	-0.0046	0.0206	0.822
rs2801008	0.0128	0.009464	0.1708	0.01537	0.02089	0.7151	0.0316	0.0222	0.155
rs2815063	9.00E-04	0.013061	0.9476	0.00058	0.02885	0.993	-0.0389	0.0301	0.1968
rs2833834	-0.0119	0.00949	0.2102	-0.02691	0.0218	0.5149	0.0243	0.0226	0.2813
rs28429256	0.013	0.00926	0.1623	0.005207	0.0208	0.9088	-0.0227	0.0209	0.2774
rs28572357	-0.005	0.008622	0.5688	-0.01263	0.01985	0.7557	-0.0221	0.0205	0.2799
rs28578714	-0.0139	0.008469	0.1001	-0.02248	0.01985	0.5548	-0.0323	0.021	0.1251

© 2023 Triozzi JL et al. *JAMA Network Open.*

rs28650790	0.0045	0.010842	0.6719	-0.00024	0.02458	0.9966	0.0034	0.03	0.909
rs28688791	-0.008	0.01051	0.4414	0.01076	0.02484	0.8376	-0.0123	0.0234	0.6003
rs28866311	-0.0431	0.007577	1.11E-09	-0.03215	0.01943	0.3649	-0.0544	0.0212	0.01011
rs2904315	-0.0198	0.00801	0.007555	-0.0192	0.02089	0.6401	-0.021	0.0252	0.4049
rs2913920	0.0125	0.010026	0.2179	0.009583	0.02303	0.8443	0.0151	0.0236	0.523701
rs2957688	0.0223	0.007577	0.002182	0.005352	0.01947	0.8994	0.0219	0.0205	0.2841
rs3098186	0.0277	0.007628	0.000269	0.04325	0.01945	0.2056	0.0538	0.0204	0.008494
rs3104552	0.0053	0.008342	0.5323	-0.02333	0.01957	0.5313	0.0156	0.0205	0.4457
rs34025993	-0.004	0.008776	0.6758	0.007057	0.01972	0.8673	-0.0095	0.0207	0.646501
rs34072724	-0.003	0.008342	0.7533	-0.00097	0.01943	0.9826	0.0083	0.0204	0.6836
rs34079867	-0.0109	0.01023	0.3074	-0.0294	0.02212	0.4791	0.0088	0.0224	0.6948
rs34130368	NA	NA	NA	0.03117	0.03038	0.597	NA	NA	NA
rs34413141	0.0072	0.011709	0.5383	-0.00566	0.02518	0.9184	-0.0066	0.0301	0.8262
rs34487963	0.0411	0.037653	0.2754	0.04187	0.07305	0.542	NA	NA	NA
rs34518929	0	0.009796	0.9665	-0.04863	0.02196	0.2076	-0.0177	0.0244	0.4688
rs34535756	0.0017	0.02227	0.9375	-0.0867	0.05112	0.3513	-0.04	0.0561	0.4762
rs34727427	0.0054	0.008903	0.5404	0.01359	0.02084	0.7492	-0.0126	0.0229	0.5827
rs34917849	0.0207	0.011097	0.04429	-0.0415	0.02913	0.4441	0.0023	0.0323	0.9425
rs34941092	-0.004	0.011633	0.7467	-0.03637	0.02722	0.4765	0.0326	0.0315	0.3004
rs35098810	-0.001	0.01023	0.96	0.01821	0.02315	0.6941	0.0074	0.0236	0.7526
rs35413927	-0.004	0.009898	0.6647	-0.01328	0.02115	0.7591	0.0061	0.0231	0.7925
rs35444	-0.009	0.008597	0.3111	-0.02822	0.01992	0.4471	0.033	0.0211	0.119
rs35680304	0.0143	0.007832	0.03973	-0.00046	0.01988	0.9919	0.0306	0.021	0.1437
rs35783704	-0.002	0.01551	0.9008	-0.02304	0.03249	0.7259	0.0264	0.0283	0.35
rs365990	-0.001	0.008648	0.9398	-0.04713	0.02009	0.1787	0.0026	0.023	0.91
rs3735533	0.0416	0.014413	0.001744	-0.01071	0.03706	0.894	0.0117	0.0351	0.739701
rs3764400	0.0118	0.012117	0.3355	0.05035	0.02904	0.3393	0.0296	0.0255	0.2469
rs3772219	0.0129	0.009056	0.1528	0.01582	0.02067	0.222028	0.0027	0.0226	0.9063
rs3802517	0.0112	0.008342	0.1822	-0.02877	0.01944	0.4243	0.0029	0.0206	0.8889
rs3807925	-0.002	0.008929	0.8088	-0.01413	0.02033	0.7317	-0.0078	0.0211	0.7117

© 2023 Triozzi JL et al. *JAMA Network Open.*

rs3815460	0.0135	0.013495	0.318	0.04058	0.03199	0.102306	0.0215	0.0412	0.6017
rs3819532	-0.0109	0.008444	0.1869	0.006925	0.01989	0.8711	0.0106	0.0205	0.6061
rs3828282	-0.0119	0.008827	0.166	-0.00831	0.01969	0.842	0.0238	0.0217	0.2717
rs3845811	-0.003	0.008648	0.7408	-0.00384	0.0196	0.9293	0.0206	0.0205	0.3146
rs3860770	0.0322	0.008342	1.21E-05	0.05543	0.02143	0.1336	0.0196	0.0208	0.3477
rs3918226	-0.0237	0.016735	0.1595	0.01141	0.03599	0.8832	-0.0537	0.0403	0.1834
rs3950627	-0.003	0.009031	0.7474	-0.0361	0.01971	0.3091	0.001	0.021	0.963
rs3980686	0.0017	0.013444	0.9019	-0.06657	0.03192	0.2391	0.0097	0.0368	0.7927
rs404100	0.0125	0.008495	0.1449	-0.0148	0.01952	0.706	0.026	0.0204	0.203
rs4143175	-0.006	0.010128	0.5552	0.02551	0.02268	0.5578	0.0079	0.0245	0.7474
rs42032	0.0066	0.009388	0.4763	-0.00015	0.02212	0.9977	-0.0574	0.0231	0.01278
rs4245599	0.0211	0.007679	0.004736	0.006691	0.01961	0.8738	0.0024	0.0204	0.9076
rs4260863	0.0122	0.008724	0.1619	0.0071	0.02002	0.8685	-0.0248	0.0221	0.2637
rs4284362	-0.0119	0.009311	0.1886	-0.04701	0.02201	0.2263	-0.024	0.0214	0.262
rs4408839	0.0163	0.00949	0.08846	-0.01295	0.02213	0.7765	0.0331	0.0228	0.1466
rs4440615	-0.0119	0.008546	0.164	-0.0306	0.02007	0.4085	-0.0322	0.0207	0.1192
rs4499560	0.0028	0.009413	0.7635	-0.02531	0.02102	0.5268	0.0173	0.0224	0.4403
rs4511593	-0.004	0.009158	0.683	0.0117	0.02038	0.7809	-0.031	0.0212	0.1433
rs4553000	-0.006	0.008393	0.4615	-0.01385	0.01938	0.7236	0.0108	0.0203	0.5947
rs4577304	0.0064	0.008444	0.4459	0.01357	0.01945	0.7306	-0.0074	0.0205	0.7188
rs4606697	-0.0139	0.018112	0.4325	-0.01999	0.03105	0.7525	-0.044	0.0372	0.2374
rs4651224	0.0147	0.007857	0.03797	-0.0031	0.01965	0.9436	0.0317	0.0205	0.1216
rs4734868	-0.0247	0.008189	0.002322	-0.01976	0.02059	0.6238	-0.018	0.0208	0.3864
rs4775769	0.0034	0.014796	0.8165	-0.0281	0.03348	0.6726	-0.055	0.0419	0.1896
rs4784541	0.0112	0.008776	0.2048	0.05399	0.01971	0.1096	0.0131	0.0206	0.5234
rs483071	0.0051	0.009209	0.5734	0.02866	0.02025	0.4475	0.0114	0.0208	0.583899
rs4834792	0.0037	0.008495	0.6593	-0.01152	0.01945	0.7735	-0.0268	0.0204	0.1896
rs4838021	-0.0208	0.013138	0.108	0.001228	0.02921	0.9853	-0.0541	0.028	0.05299
rs4873492	0.011	0.011658	0.3449	0.002529	0.02593	0.9655	0.0399	0.0273	0.1434
rs4876133	0.0114	0.009311	0.2202	-0.00799	0.02155	0.8622	0.0255	0.0233	0.2722

© 2023 Triozzi JL et al. *JAMA Network Open.*

rs488834	-0.01	0.011327	0.3649	-0.03583	0.02287	0.3942	-0.0221	0.0237	0.3504
rs4888408	-0.0296	0.007679	3.43E-05	-0.04758	0.01977	0.1663	-0.0547	0.0206	0.007934
rs4925159	-0.0159	0.008342	0.05323	-0.0189	0.01958	0.6215	-0.0141	0.0205	0.4909
rs4926499	-0.0247	0.012628	0.01787	0.009223	0.0262	0.8695	7.00E-04	0.0259	0.9799
rs4932373	0.0033	0.009821	0.7346	-0.01567	0.0207	0.7062	0.0168	0.023	0.466
rs4952609	0.0267	0.009439	0.000836	0.03214	0.02266	0.4463	-0.0029	0.022	0.8935
rs4955575	-0.0237	0.008724	0.006195	-0.03456	0.02244	0.4032	0.021	0.0223	0.3463
rs4957026	0.0062	0.008801	0.4851	-0.02489	0.02064	0.526	0.0039	0.0218	0.8571
rs4961293	-0.005	0.008673	0.5782	0.01042	0.01952	0.7972	0.0095	0.0204	0.643301
rs4980379	0.0065	0.008852	0.4574	0.01432	0.02022	0.7264	0.0177	0.0208	0.3947
rs509564	-0.0237	0.008954	0.006781	-0.0335	0.02367	0.4474	0.0038	0.0227	0.8681
rs509833	0.0038	0.0125	0.7632	-0.04499	0.02774	0.3755	-0.0326	0.0312	0.2952
rs55732192	0.0094	0.014847	0.5205	-0.02698	0.03343	0.6858	-0.0034	0.0308	0.913
rs55924432	-0.001	0.00875	0.9474	0.03049	0.01984	0.4045	0.0231	0.0205	0.2589
rs55944332	-0.0237	0.009209	0.01276	-0.03698	0.02293	0.3786	0.0167	0.0234	0.4771
rs56288724	-0.0149	0.007781	0.02396	0.01276	0.01963	0.75	0.0236	0.0209	0.2586
rs56407827	-0.007	0.00977	0.5038	-0.02931	0.022	0.4781	0.014	0.022	0.5262
rs571689	0.0294	0.007449	2.78E-05	-0.00332	0.01952	0.9389	0.0164	0.0207	0.4276
rs573455	0.0028	0.008418	0.7382	-0.00136	0.01942	0.996	-0.028	0.0204	0.1716
rs5742643	-0.005	0.010179	0.6459	-0.04575	0.0224	0.2501	0.0013	0.024	0.9562
rs57786342	-0.01	0.010459	0.3389	0.01156	0.02421	0.82	0.0288	0.0269	0.2852
rs57866767	0.0106	0.008469	0.2109	-0.00604	0.01959	0.8865	0.0032	0.0214	0.8823
rs57946343	0.0046	0.01199	0.7028	0.02886	0.02706	0.5809	-0.0045	0.0335	0.8929
rs59980837	0.0948	0.027526	0.000372	0.1904	0.07308	0.1303	0.0891	0.0916	0.331
rs60138042	0.0013	0.017755	0.9427	-0.04219	0.04017	0.5873	-0.0223	0.0383	0.56
rs60191654	-0.0119	0.01074	0.2837	-0.00572	0.02475	0.9161	0.0399	0.0274	0.1449
rs6026578	0.0228	0.008087	0.002746	-0.00157	0.02015	0.9725	0.0348	0.0213	0.1025
rs6026744	-0.0247	0.011684	0.03717	0.02492	0.03005	0.6768	0.0058	0.0274	0.8311
rs6029756	-0.009	0.009056	0.3295	-0.03397	0.02105	0.3782	-0.0142	0.021	0.4979
rs6031431	-0.003	0.00875	0.6888	-0.00937	0.01957	0.8194	-0.0332	0.0204	0.1044

© 2023 Triozzi JL et al. *JAMA Network Open.*

rs60444686	2.00E-04	0.021505	0.993	0.01197	0.05138	0.9154	0.0043	0.0542	0.9369
rs604723	-0.004	0.009592	0.6726	-0.00664	0.02187	0.8883	0.011	0.0233	0.6369
rs6054139	-0.007	0.008776	0.4016	-0.00129	0.0198	0.9771	0.0299	0.0211	0.1562
rs6058088	-0.0159	0.01125	0.1747	0.01532	0.02746	0.7876	0.0156	0.0253	0.5383
rs6062324	0.0208	0.009082	0.01763	0.03087	0.02292	0.4725	-8.00E-04	0.0249	0.9759
rs6078093	0.0105	0.00852	0.2246	-0.02914	0.01965	0.4232	1.00E-04	0.0207	0.9979
rs6090907	0.0168	0.012679	0.1829	0.01491	0.02729	0.7922	-0.0104	0.0353	0.766999
rs60909079	-0.0178	0.009643	0.07078	0.0108	0.02218	0.816	1.00E-04	0.0252	0.9984
rs60991988	-0.009	0.014235	0.5469	-0.00199	0.03196	0.9781	0.0304	0.0352	0.3879
rs6108787	0.0034	0.008367	0.6808	0.0554	0.01947	0.095	-0.0011	0.0206	0.9582
rs61772592	0.0023	0.013265	0.8594	0.0759	0.02964	0.1379	-0.0704	0.0273	0.009943
rs61917655	-0.0198	0.014413	0.1765	-0.00207	0.03354	0.9784	0.0624	0.0346	0.071661
rs62047964	-0.0344	0.019362	0.07626	-0.0936	0.04152	0.1987	-0.0042	0.0425	0.9213
rs62076622	-0.009	0.011148	0.4414	-0.02286	0.02443	0.6334	0.0066	0.0242	0.7845
rs62082230	0.0276	0.008801	0.001832	0.004786	0.0218	0.9205	2.00E-04	0.0251	0.9947
rs62170470	0.008	0.009184	0.3841	0.01155	0.01998	0.7793	-0.0046	0.0206	0.8251
rs62309747	0.0072	0.00875	0.4013	-0.00246	0.01943	0.955	0.0211	0.0203	0.299
rs62512914	-0.003	0.008597	0.7245	-0.05004	0.01968	0.1409	-0.0106	0.0205	0.6052
rs6271	0.0145	0.016046	0.3682	0.04453	0.03688	0.5254	0.0092	0.041	0.8218
rs641620	-0.0129	0.012296	0.2856	-0.00261	0.02835	0.9674	-0.0237	0.0283	0.4029
rs6438857	0.0184	0.007755	0.006134	-0.00303	0.01972	0.945	0.0334	0.0205	0.1035
rs6445583	-0.0119	0.009796	0.2111	0.01054	0.02221	0.821	-4.00E-04	0.0236	0.9875
rs6452769	-0.0188	0.00926	0.02317	-0.03216	0.02443	0.4839	-0.0379	0.0294	0.1976
rs6490019	-0.003	0.008622	0.7306	0.001746	0.02004	0.9692	-0.0121	0.0222	0.586001
rs6504213	0.0053	0.008827	0.5455	-0.02196	0.01977	0.5632	0.0023	0.0206	0.9115
rs6540119	0.0228	0.008776	0.01335	0.02193	0.02087	0.5872	0.0077	0.0207	0.708401
rs6562778	-0.0208	0.007755	0.00395	-0.03801	0.01953	0.2761	-0.0029	0.0205	0.8865
rs658780	0.0155	0.008546	0.04183	0.002258	0.0223	0.9641	0.0131	0.0247	0.5955
rs665445	0.015	0.009235	0.1042	0.009765	0.02159	0.83	0.011	0.0239	0.647
rs66864335	0.016	0.010153	0.1123	0.02304	0.02324	0.6109	0.0273	0.0257	0.2888

© 2023 Triozzi JL et al. *JAMA Network Open.*

rs6699618	-0.0129	0.011556	0.2744	-0.04619	0.02625	0.3312	-0.0188	0.029	0.5155
rs6731373	-0.007	0.00926	0.4723	-0.05198	0.02023	0.1364	-0.0476	0.0245	0.05209
rs6732123	0.0026	0.008495	0.7627	-0.00046	0.01977	0.9919	-0.0082	0.0211	0.6955
rs6737318	0.0098	0.010179	0.3354	-0.02455	0.02354	0.5903	0.0014	0.0247	0.954
rs67617547	-0.008	0.009005	0.3715	-0.02413	0.02057	0.5389	-0.0131	0.022	0.5496
rs6771917	-0.003	0.010102	0.7835	-0.00868	0.02276	0.858	-0.0219	0.0254	0.388
rs67885470	-0.0198	0.011531	0.07628	-0.02368	0.02458	0.6223	NA	NA	NA
rs6788907	-0.003	0.009566	0.7638	-0.00821	0.02181	0.8599	-0.0135	0.0229	0.555199
rs6788984	0.0113	0.01227	0.354	0.002306	0.02784	0.9708	0.0477	0.0385	0.2162
rs68085857	0.0113	0.009923	0.2575	-0.01671	0.02299	0.7187	-0.0017	0.024	0.9439
rs68115553	-0.0178	0.032551	0.5821	-0.03581	0.07283	0.8142	-2.00E-04	0.0635	0.9976
rs6870654	-0.005	0.009643	0.611	-0.03199	0.02225	0.4392	-0.0285	0.0257	0.2661
rs6892983	0.0197	0.007679	0.008903	0.009796	0.01978	0.8128	0.0221	0.0215	0.3041
rs6921291	-0.009	0.011556	0.4414	-0.02335	0.02482	0.6313	-0.0328	0.0309	0.2877
rs6957161	0.0028	0.009923	0.7769	0.01621	0.02202	0.7148	0.0051	0.0248	0.8384
rs6961048	0.0187	0.013648	0.1745	-0.00347	0.03222	0.9618	0.0051	0.0299	0.8647
rs6986368	0.0025	0.009337	0.7852	-0.00888	0.02087	0.8406	-0.0108	0.0229	0.6364
rs7012866	0.0091	0.008316	0.2787	0.001619	0.01941	0.9706	-0.007	0.0209	0.736999
rs702395	0.0031	0.008546	0.717	-0.02751	0.01959	0.4515	0.0176	0.0204	0.3882
rs7026176	0.0186	0.007653	0.008856	0.01555	0.01941	0.6883	-0.0018	0.0204	0.9283
rs7045409	0.0102	0.008776	0.2484	-0.02656	0.02016	0.4837	0.0398	0.0222	0.073639
rs708117	-0.009	0.008571	0.3005	-0.02527	0.01943	0.4899	-0.0196	0.0206	0.3399
rs7093894	0.0057	0.012577	0.6533	-0.01715	0.02753	0.7611	0.0184	0.0337	0.5852
rs7107356	0.0027	0.008418	0.7445	-0.04306	0.01938	0.206	0.0088	0.0209	0.674401
rs7134440	0.0038	0.015638	0.8068	-0.02084	0.0353	0.7744	-0.0441	0.0357	0.216
rs7134677	0.0082	0.009898	0.4098	0.01254	0.02125	0.7745	0.0192	0.021	0.3626
rs7154723	0.0057	0.008929	0.5191	0.02934	0.01999	0.4287	0.0209	0.0212	0.3248
rs7186298	0.0172	0.007551	0.01498	0.02475	0.01956	0.5031	-0.0083	0.021	0.6909
rs7198817	-0.0169	0.008673	0.05902	-0.00559	0.02014	0.8984	-0.0185	0.0206	0.3711
rs7211535	-0.01	0.00875	0.236	-0.00735	0.01945	0.8594	-0.0032	0.0205	0.8754

© 2023 Triozzi JL et al. *JAMA Network Open.*

rs7213273	0.0354	0.007934	1.18E-06	0.0582	0.0204	0.0942	0.0278	0.0214	0.1934
rs7236548	0.0173	0.010893	0.1134	-0.04559	0.0252	0.316	0.0122	0.0242	0.6138
rs7255933	0	0.00949	0.9795	0.06459	0.02217	0.0866	-0.0043	0.0224	0.8468
rs72683923	-0.0535	0.036097	0.134	0.004768	0.06938	0.9758	-0.0431	0.0978	0.6598
rs72719160	-0.006	0.009133	0.5242	0.008326	0.02091	0.8515	0.0202	0.0225	0.3708
rs72742507	-0.0188	0.008265	0.02449	-0.06209	0.02124	0.0854	0.0237	0.0219	0.2803
rs7278003	-0.008	0.008469	0.3326	0.02617	0.01953	0.475	0.0025	0.022	0.9106
rs72847885	-0.01	0.008776	0.2804	-0.00472	0.02051	0.9165	-0.0137	0.0215	0.5246
rs72931748	-0.0257	0.015408	0.1022	0.02611	0.03419	0.7036	-0.0124	0.0243	0.6106
rs73046792	0.0093	0.013265	0.4774	-0.00082	0.02615	0.9891	0.0733	0.0326	0.02446
rs73049928	-0.002	0.011352	0.8402	0.02467	0.02475	0.6087	0	0.0225	0.999
rs7306710	0.0055	0.008418	0.5199	0.01137	0.01956	0.778	0.0408	0.0205	0.04613
rs73075659	-0.005	0.009337	0.5582	0.01227	0.02045	0.7704	-0.035	0.0243	0.1498
rs73103937	-0.0129	0.009898	0.1852	-0.00298	0.02202	0.9517	-0.0341	0.024	0.1563
rs7310615	0.0112	0.008367	0.1851	0.02676	0.01954	0.4642	0.0198	0.0207	0.3379
rs7331680	-0.0119	0.01176	0.3274	-0.00894	0.02728	0.8791	-0.0175	0.0285	0.5394
rs73727605	-0.0334	0.019005	0.08065	-0.06846	0.03886	0.3305	-0.0969	0.049	0.04797
rs73855810	-0.005	0.012168	0.6918	-0.02967	0.02792	0.5823	-0.0613	0.0307	0.04593
rs7395791	-0.002	0.008546	0.8125	-0.06482	0.01956	0.048	-0.0207	0.0205	0.312
rs74048190	0.0034	0.020383	0.8669	0.054	0.04632	0.5417	-0.0275	0.0438	0.5297
rs740746	-0.009	0.009388	0.327	0.02398	0.02205	0.5725	-0.0353	0.0234	0.1323
rs743395	0.0063	0.008903	0.4817	0.01964	0.02039	0.6222	-0.0143	0.0208	0.4935
rs7439567	-0.008	0.008878	0.3605	-0.02224	0.01981	0.5584	0.0201	0.0205	0.3273
rs74538877	0.0239	0.019362	0.2198	0.01954	0.04346	0.831	6.00E-04	0.0425	0.9892
rs7463212	-0.0227	0.007474	0.001151	-0.03856	0.01948	0.2669	0.0379	0.0206	0.065321
rs7491248	-0.0247	0.009209	0.002528	0.001431	0.02338	0.9785	-0.0134	0.0292	0.645601
rs7493678	0.0105	0.009005	0.2489	0.01378	0.02035	0.7388	1.00E-04	0.0215	0.9978
rs75016974	-0.0178	0.012372	0.1481	-0.01974	0.02779	0.7253	-0.0092	0.0301	0.7596
rs7514579	0.0011	0.010102	0.9115	-0.05745	0.02303	0.1496	-0.0615	0.025	0.0138
rs75461554	0.0147	0.010485	0.1592	0.01368	0.02441	0.7865	0.007	0.0248	0.778501

© 2023 Triozzi JL et al. *JAMA Network Open.*

rs7555285	0.005	0.010561	0.6287	-0.03754	0.02449	0.4059	-0.0412	0.0239	0.08567
rs75672964	0.0056	0.022347	0.801	0.02236	0.04918	0.829	0.0945	0.0388	0.01479
rs75961402	-0.001	0.011505	0.9265	0.003158	0.02679	0.9581	-0.0093	0.0304	0.759899
rs7615099	-0.001	0.00898	0.8915	0.00165	0.02064	0.9718	0.0154	0.0218	0.4796
rs7618284	0.0227	0.00852	0.007148	0.01658	0.0209	0.6915	-0.0337	0.024	0.1597
rs76443575	0	0.022398	0.9929	0.04675	0.05254	0.6521	-0.0026	0.0512	0.9589
rs76452347	6.00E-04	0.010332	0.9566	0.03452	0.02492	0.4585	0.0269	0.0267	0.3127
rs76719272	0.0179	0.013291	0.1819	0.006499	0.02894	0.9185	0.0268	0.03	0.3709
rs7683728	0.0136	0.008444	0.1087	-0.01855	0.01947	0.6265	-0.0238	0.0209	0.2541
rs7703560	0.0109	0.00949	0.2458	-0.00012	0.02134	0.998	0.0017	0.0227	0.9392
rs7722243	-0.01	0.008597	0.2615	-0.01145	0.01947	0.7751	-0.0092	0.0205	0.652501
rs7725413	0.0111	0.010281	0.2812	0.00856	0.02299	0.8617	0.0065	0.0242	0.7887
rs77375686	0.0104	0.014413	0.4769	0.06854	0.03119	0.2116	-0.0062	0.0359	0.8632
rs7744902	-0.0402	0.014464	0.008978	-0.06642	0.03689	0.3185	0.0395	0.0396	0.3175
rs7763558	8.00E-04	0.009056	0.9259	-0.01172	0.02078	0.785	0.0022	0.0221	0.9216
rs7765526	-0.0119	0.008699	0.1859	-0.03266	0.01966	0.3628	-9.00E-04	0.0205	0.9649
rs778124	0.0075	0.008827	0.3851	-0.01927	0.02007	0.6236	0.0198	0.0218	0.3647
rs77924615	0.1828	0.00949	1.12E-92	0.1927	0.02486	1.00E-200	0.0421	0.0247	0.08749
rs7796	0.0038	0.009158	0.6797	-0.01339	0.0194	0.7337	-0.0286	0.0205	0.1635
rs7821832	-0.0149	0.009847	0.1273	-0.00438	0.0223	0.9291	9.00E-04	0.0223	0.9674
rs7844887	0.0179	0.009005	0.0264	0.005016	0.02335	0.9222	0.0307	0.0239	0.1991
rs78474310	0.0373	0.021046	0.07559	0.01483	0.04614	0.8815	0.0497	0.0506	0.3258
rs78648104	-0.0237	0.014821	0.1117	0.0663	0.03464	0.2851	1.00E-04	0.0278	0.9979
rs786923	-0.0149	0.008954	0.09485	-0.05434	0.01994	0.1118	-0.0606	0.0224	0.006902
rs78998485	0.0091	0.009694	0.3415	-0.0028	0.02214	0.9549	-0.0087	0.0219	0.6904
rs79069610	-0.0119	0.02	0.538	-0.06756	0.04553	0.4229	-0.034	0.0628	0.5886
rs7912283	-0.0139	0.009821	0.1504	-0.00623	0.02036	0.8875	0.0035	0.0211	0.8671
rs7926110	-0.004	0.009056	0.6567	0.01166	0.02075	0.7858	-0.0135	0.0238	0.57
rs79384779	0.0103	0.012372	0.4063	0.009486	0.02774	0.8735	0.0227	0.0259	0.3822
rs79539362	-0.0139	0.014158	0.315	0.02057	0.03178	0.7511	0.0111	0.036	0.757

© 2023 Triozzi JL et al. *JAMA Network Open.*

rs7963801	-0.0119	0.009031	0.1901	0.007173	0.0197	0.8648	-0.0071	0.0207	0.733301
rs79782817	-0.0218	0.013571	0.1018	-0.01718	0.03252	0.7996	-0.0298	0.0336	0.3748
rs7980644	0.0049	0.011709	0.6772	-0.0084	0.02644	0.8829	0.0363	0.0282	0.1979
rs79930761	-0.0266	0.016505	0.1042	0.002172	0.03493	0.9782	0.0304	0.0468	0.5162
rs8003103	0.0205	0.007959	0.007193	0.02534	0.02071	0.5191	-0.0144	0.0219	0.5088
rs8030856	0.0122	0.008699	0.1597	0.001119	0.02004	0.9804	0.0048	0.0206	0.8145
rs8044992	0.005	0.00926	0.5955	-0.01359	0.02148	0.7571	-0.0082	0.0234	0.726599
rs8054587	1.00E-04	0.008546	0.9943	0.01668	0.01944	0.665	1.00E-04	0.0215	0.9959
rs8079811	0.0162	0.008087	0.0349	0.02043	0.02054	0.6096	0.0243	0.0206	0.2382
rs8113613	-0.0325	0.009872	0.000138	-0.04387	0.02487	0.3299	NA	NA	NA
rs8125763	0.0065	0.008776	0.4535	0.01081	0.01952	0.7891	0.016	0.0205	0.4345
rs8142376	0.015	0.008265	0.07278	0.03061	0.01939	0.39	-0.0095	0.0205	0.6434
rs8180684	-0.0178	0.009337	0.05325	-0.01642	0.02139	0.702	-0.0095	0.0224	0.6697
rs848445	0.0313	0.00875	6.36E-05	-0.01191	0.0214	0.7881	9.00E-04	0.0233	0.9706
rs869396	0.0244	0.007781	0.000789	0.04616	0.01959	0.1768	-0.0083	0.0206	0.6875
rs871004	0.0128	0.009031	0.1575	-0.0067	0.02047	0.8791	0.0312	0.0206	0.1301
rs8904	0.0113	0.008673	0.188	0.007619	0.02015	0.8593	0.0343	0.0209	0.1016
rs908951	-0.0218	0.00773	0.00195	-0.08489	0.01976	0.0085	-0.0192	0.0208	0.3555
rs927315	0.0241	0.00801	0.002491	0.01863	0.01956	0.6265	0.0051	0.0207	0.8048
rs9285476	-0.0188	0.009719	0.06066	-0.05946	0.02132	0.1026	-0.019	0.0241	0.43
rs9302885	0.0052	0.008597	0.5413	0.006271	0.01951	0.8815	0.0175	0.0211	0.405
rs9327297	0.0029	0.009031	0.7441	-0.00313	0.02063	0.9458	0.0132	0.0219	0.5471
rs9349379	-0.009	0.00852	0.2942	0.06602	0.01975	0.0459	0.003	0.0204	0.8842
rs9361836	8.00E-04	0.009082	0.9289	-0.03335	0.02096	0.3858	-0.0098	0.0216	0.649201
rs9368222	0.0035	0.009337	0.7104	-0.01642	0.02205	0.7114	0.0339	0.0217	0.1184
rs9401913	0.0225	0.007577	0.001025	0.01474	0.01953	0.7072	0.0729	0.0204	0.000343
rs9486916	0.0084	0.010408	0.4175	0.02139	0.02502	0.6664	-0.0489	0.0245	0.0462
rs9507885	-0.002	0.014898	0.8737	-0.03059	0.03399	0.648	0.0821	0.0335	0.01413
rs9508495	-0.01	0.009898	0.3052	-0.03146	0.0229	0.4626	-0.043	0.0212	0.04266
rs9526707	0.0102	0.009337	0.2811	-0.0169	0.02069	0.6818	0.0247	0.0218	0.2577

© 2023 Triozzi JL et al. *JAMA Network Open.*

rs9549627	0.0296	0.012066	0.01042	-0.00394	0.03062	0.9541	0.0348	0.0327	0.2868
rs961764	0.0108	0.008444	0.2002	0.005252	0.01961	0.9021	0.0175	0.0207	0.3972
rs9848170	0.0085	0.00852	0.3256	-0.01524	0.01985	0.7018	0.003	0.0204	0.8843
rs9869437	0.0165	0.007959	0.03995	-0.00025	0.02046	0.9957	0.002	0.0219	0.9283
rs9876694	-0.0325	0.016556	0.0246	-0.01373	0.04133	0.8772	-0.0169	0.0469	0.7179
rs9880098	-0.008	0.008776	0.3435	-0.00158	0.01994	0.9721	-0.0061	0.0204	0.764599
rs9886665	-0.01	0.009541	0.2967	0.01517	0.02223	0.7368	8.00E-04	0.0224	0.9711
rs9897429	-0.004	0.008852	0.6242	-0.02675	0.01972	0.469	0.0284	0.0206	0.1683
rs9899540	0.0141	0.00875	0.1096	-0.02832	0.02008	0.4492	0.0046	0.0214	0.8296
rs9918876	1.00E-04	0.014082	0.9932	0.02157	0.03259	0.7451	0.0383	0.0289	0.1845

Abbreviation: MVP, Million Veteran Program; UKB, UK Biobank; FinnGen, FinnGen Study; SNP, single nucleotide polymorphism; se, standard error.

© 2023 Triozzi JL et al. *JAMA Network Open.*

eTable 9. The inverse variance-weighted effect of genetic proxies of thiazide diuretics, beta blockers, and systolic blood pressure on kidney stones in the Million Veteran Program, UK Biobank, and FinnGen study.

Exp	Study	nsnp	beta[a]	se	pval
TZ	MVP	4	-0.16855	0.027963	1.66E-09
TZ	UKB	4	-0.10023	0.070499	0.155119
TZ	FinnGen	4	-0.18324	0.067124	0.006336
BB	MVP	20	0.032793	0.008245	6.97E-05
BB	UKB	19	-0.04388	0.019405	0.023758
BB	FinnGen	20	0.054028	0.020333	0.007879
SBP	MVP	439	0.002399	0.002932	0.413243
SBP	UKB	392	0.002826	0.005038	0.574833
SBP	FinnGen	433	-0.00114	0.004051	0.777787

Abbreviations: Exp, exposure; TZ, thiazide; BB, beta blocker; SBP, systolic blood pressure; MVP, Million Veteran Program; UKB, UK Biobank; FinnGen, FinnGen study; nsnp, number of single nucleotide polymorphisms; se, standard error.

[a] The effect estimates for genetic proxies of thiazide diuretics and beta blockers are inverted to reflect the negative relationship between the drug effect and blood pressure.

© 2023 Triozzi JL et al. *JAMA Network Open.*

eTable 10. The combined random-effects model meta-analysis of kidney stone risk in the Million Veteran Program, UK Biobank, and FinnGen study.

Exp	OR[a]	OR lower CI	OR upper CI	zval	pval	tau2	tau2 lower CI	tau2 upper CI	I2	I2 lower CI	I2 upper CI	H	H lower CI	H upper CI	Q	Qdf	Qp
TZ	0.85	0.81	0.89	-6.70	0.00	0.00	0.00	0.07	0.00	0.00	0.90	1.00	1.00	3.10	0.92	2.00	0.63
BB	1.02	0.96	1.07	0.52	0.60	0.00	0.00	0.10	0.87	0.63	0.96	2.79	1.65	4.70	15.53	2.00	0.00
SBP	1.00	1.00	1.01	0.69	0.49	0.00	0.00	0.00	0.00	0.00	0.90	1.00	1.00	3.10	0.59	2.00	0.75

Abbreviations: Exp, exposure; TZ, thiazide; BB, beta blocker; SBP, systolic blood pressure; MVP, Million Veteran Program; UKB, UK Biobank; FinnGen, FinnGen study; OR, odds ratio; CI, confidence interval.

[a] The effect estimates for genetic proxies of thiazide diuretics and beta blockers are inverted to reflect the negative relationship between the drug effects and blood pressure.

© 2023 Triozzi JL et al. *JAMA Network Open.*

eTable 11. Heterogeneity and pleiotropy testing for the main analysis and negative controls (Cochran's Q, MR-Egger Q, MR-Egger intercept, and MR-PRESSO tests).

Exposure	Study	IVW Q	IVW Q df	IVW Q pval	Egger Q	Egger Q df	Egger Q pval	Egger Intercept	Egger Intercept se	Egger Intercept pval	MRPRESSO GlobalTest RSSobs	MRPRESSO GlobalTest Pvalue
TZ	MVP	1.5453	3	0.6719	1.0353	2	0.5959	0.0107	0.0150	0.5492	2.7788	0.782
TZ	UKB	2.2062	3	0.5307	0.8501	2	0.6537	0.0437	0.0376	0.3643	3.7515	0.597
TZ	Finn Gen	1.2982	3	0.7295	0.4665	2	0.7919	0.0327	0.0359	0.4580	2.1977	0.787
BB	MVP	15.8477	19	0.6674	15.68392	18	0.6146	-0.0026	0.0063	0.6905	18.1199	0.724
BB	UKB	11.1236	18	0.8890	10.5430	17	0.8793	-0.0121	0.0158	0.4565	12.1617	0.946
BB	Finn Gen	9.2212	19	0.9696	7.325444	18	0.9871	0.0219	0.0159	0.1854	10.8742	0.966
SBP	MVP	1637.24	438	0.0000	1637.017	437	0.0000	-0.0006	0.00225	0.8066	1680.6310	<0.001
SBP	UKB	696.692	391	0.0000	695.1184	695	0.0000	0.0000	0.0037	0.0040	772.7067	<0.001
SBP	Finn Gen	501.923	432	0.0112	500.2997	431	0.0117	0.0037	0.0040	0.3480	522.0000	0.012

Abbreviations: TZ, thiazide; BB, beta blocker; SBP, systolic blood pressure; MVP, Million Veteran Program; UKB, UK Biobank; FinnGen, FinnGen study; nsnp, number of single nucleotide polymorphisms; se, standard error.

© 2023 Triozzi JL et al. *JAMA Network Open.*

eTable 12. Sensitivity analyses for the main analyses and negative controls (weighted median, weighted mode, and multiplicative random-effects inverse variance weighted effect estimates at multiple clumping thresholds).

Study	Method	TZ nsnp	TZ beta[a]	TZ se	TZ pval	BB nsnp	BB beta[a]	BB se	BB pval	SBP nsnp	SBP beta	SBP se	SBP pval
MVP	Weighted median	4	-0.15827	0.039901	7.29E-05	20	-0.03619	0.011345	0.001425	439	0.00364	0.002826	0.19764
MVP	Weighted mode	4	-0.15101	0.046781	0.048288	20	-0.03438	0.012584	0.013249	439	0.006636	0.006139	0.280309
MVP	IVW r2 = 0.2	3	-0.16658	0.031149	8.90E-08	14	-0.02424	0.010865	0.025648	NA	NA	NA	NA
MVP	IVW r2 = 0.1	3	-0.17434	0.032243	6.41E-08	12	-0.01591	0.011536	0.167905	NA	NA	NA	NA
MVP	IVW r2 = 0.05	2	-0.15424	0.037447	3.81E-05	11	-0.00786	0.019841	0.691819	NA	NA	NA	NA
MVP	IVW r2 = 0.01	2	-0.16992	0.046749	0.000278	8	-0.01664	0.015022	0.267942	NA	NA	NA	NA
UKB	Weighted median	4	-0.04522	0.086476	0.601065	19	0.056178	0.026473	0.033831	392	-0.00409	0.006438	0.52475
UKB	Weighted mode	4	-0.03452	0.100352	0.753537	19	0.060237	0.0279	0.044592	392	-0.01798	0.011395	0.115371
UKB	IVW r2 = 0.2	3	-0.08627	0.07937	0.277061	13	0.032238	0.025535	0.20677	NA	NA	NA	NA
UKB	IVW r2 = 0.1	3	-0.1417	0.081137	0.08074	12	0.048107	0.0265	0.069466	NA	NA	NA	NA
UKB	IVW r2 = 0.05	3	-0.08627	0.07937	0.277061	10	0.033292	0.037114	0.369719	NA	NA	NA	NA
UKB	IVW r2 = 0.01	2	-0.12722	0.123172	0.301661	8	0.021168	0.032554	0.515546	NA	NA	NA	NA
Finn Gen	Weighted median	4	-0.14268	0.085485	0.095111	20	-0.06568	0.026014	0.011582	433	0.003216	0.006151	0.601063
Finn Gen	Weighted mode	4	-0.13886	0.093793	0.235328	20	-0.07134	0.029499	0.025807	433	0.006353	0.013983	0.649814
Finn Gen	IVW r2 = 0.2	3	-0.17077	0.074489	0.021874	14	-0.03291	0.026512	0.21443	NA	NA	NA	NA
Finn Gen	IVW r2 = 0.1	3	-0.18312	0.081097	0.023942	12	-0.04486	0.027804	0.106613	NA	NA	NA	NA
Finn Gen	IVW r2 = 0.05	2	-0.20872	0.099165	0.035313	11	-0.04043	0.038956	0.299309	NA	NA	NA	NA

© 2023 Triozzi JL et al. *JAMA Network Open.*

Finn Gen	IVW r2 = 0.01	2	-0.13884	0.092298	0.132507	8	-0.04297	0.033731	0.202727	NA	NA	NA	NA

Abbreviations: TZ, thiazide; BB, beta blocker; SBP, systolic blood pressure; MVP, Million Veteran Program; UKB, UK Biobank; FinnGen, FinnGen study; nsnp, number of single nucleotide polymorphisms; IVW, inverse variance-weighted; se, standard error.

[a] The effect estimates for genetic proxies of thiazide diuretics and beta blockers are inverted to reflect the negative relationship between the drug effect and blood pressure.

© 2023 Triozzi JL et al. *JAMA Network Open.*

eTable 13. The inverse variance-weighted effect of genetic proxies of thiazide diuretics on serum laboratory values.

Outcome	beta[a]	se	pval
A1c	-0.0094	0.01335	0.48133
Albumin	0.011087	0.00893	0.214402
Alkaline phosphatase	-0.0094	0.01335	0.48133
Calcium	0.05137	0.00925	2.8E-08
Cholesterol	0.065493	0.01512	1.48E-05
Glucose	-0.00168	0.009016	0.851979
Potassium	-0.07296	0.021595	0.000729
Phosphorus	-0.00087	0.008828	0.921152
Urate	0.000977	0.007371	0.894555
Vitamin D	-0.00681	0.009571	0.476594

[a] The effect estimates are inverted to reflect the negative relationship between the drug effect and blood pressure.

© 2023 Triozzi JL et al. *JAMA Network Open.*

eMethods. Genome-wide PheWAS methods from Million Veteran Program.

Million Veteran Program (MVP)
The VA Million Veteran Program (MVP) is a national cohort launched in 2011 designed to study the contributions of genetics, lifestyle, and military exposures to health and disease among US Veterans.[1] Blood biospecimens were collected for DNA isolation and genotyping, and the biorepository was linked with the VA EHR, which includes diagnosis codes (International Classification of Diseases ninth revision [ICD-9] and tenth revision [ICD-10]), laboratory measures, and detailed survey questionnaires collected at the time of enrollment for all Veterans followed in the healthcare system up to September 2019.

Genotyping, Quality Control, and Imputation
Specimen collection and genotype quality control have been described in detail before.[2,3] In brief, blood specimens were collected at recruitment sites across the country then shipped within 24 hours to the VA Central Biorepository in Boston, MA for processing and storage. Study participants were genotyped using a customized Affymetrix Axiom biobank array (the MVP 1.0 Genotyping Array), containing over 730,000 variants. Duplicate samples were excluded as well as samples with observed heterozygosity greater than the expected heterozygosity, missing genotype call rate greater than 2.5%, or incongruence between sex inferred from genetic information and gender extracted from phenotype data. Probes with high missingness (>20%), those that were monomorphic, or those with a Hardy Weinberg Equilibrium p<1E-06 in both the overall cohort and within one of the 3 major HARE groups (non-Hispanic White, non-Hispanic Black, or Hispanic/Latino). See below for HARE methods. Population-specific principal components (PCs) were computed using PLINK.[4] Genetic imputation was performed to a hybrid imputation panel comprised of the African Genome Resources panel (https://imputation.sanger.ac.uk/?about=1#referencepanels) and 1000G p3v5[5] using SHAPEIT4 (v 4.1.3)[6], and Minimac4[7].

Ancestry assignment
The harmonized race/ethnicity and genetic ancestry (HARE) approach, developed by MVP, was used to assign individuals to ancestral groups.[8] This machine learning algorithm leverages information from both the self-reported race/ethnicity data from the MVP Baseline survey and genotype data to categorized Veterans into four mutually exclusive groups: (1) non-Hispanic White (EUR), (2) non-Hispanic Black (AFR), (3) Hispanic or Latino (HIS), or (4) Asian (ASN).

Phenotype Data
EHR-derived clinical outcomes (PheCodes)
The clinical outcome from EHR was defined by phecodes curated by the MVP Data Core.[9] Each phecode represents ICD codes grouped into clinically relevant phenotypes for clinical studies. Using this approach, all ICD codes for all Veterans in MVP were extracted and each assigned a phenotype defined by a phecode. ICD-9 and ICD-10 codes were mapped to 1,876 phecodes, as previously described.[10] For each phecode, participants with ≥2 phecode-mapped ICD-9 or ICD-10 codes were defined as cases, whereas those with no instance of a phecode-mapped ICD-9 or ICD-10 code were defined as controls. Based on our previous simulation studies of ICD EHR data, populations where the phecode comprises < 200 cases or controls were more likely to result in spurious results, and we thus applied this threshold in each of the four HARE-defined ancestry groups.

Laboratory measurements
For quantitative traits, we calculated the minimum, maximum, and mean value across all visits for each participant and analyzed each resulting phenotype. Only quantitative traits with data for more than 1000 individuals within each HARE-defined ancestry group were included in the analyses. The remaining 69 laboratory measurements that passed quality control were normalized using a rank-based inverse-normal transformation. We additionally filtered values greater than six standard deviations from the mean to remove extreme outliers.

Survey Questions
The two surveys (questionnaires) for MVP, as noted previously, were designed to augment data that are contained in the electronic health record of each participant.[1] As with other study activities and all study materials sent to participants, these documents were approved by the VA Central IRB. As participants are

© 2023 Triozzi JL et al. *JAMA Network Open.*

enrolled, informed consent and HIPAA authorization forms are scanned by field site staff and sent to the CERC, to be checked for accuracy and completeness, and the data are entered in GenISIS.

Conceptually, the MVP Baseline Survey was designed to collect information regarding demographics, family pedigree, health status, lifestyle habits, military experience, medical history, family history of specific illnesses, and physical features. The MVP Lifestyle Survey contains questions from validated instruments in domains selected to provide information on sleep and exercise habits, environmental exposures, dietary habits, and sense of well-being.

Genetic association analyses

Within each HARE-defined ancestry group (AFR, ASN, EUR, HIS), genetic variants were tested for their association with the trait of interest using generalized linear mixed models to account for participant relatedness using a GPU-optimized version of the SAIGE package[11] implemented on the U.S. Department of Energy Summit supercomputer. Directly genotyped variants were used for step 1 of SAIGE. LD-based variant pruning was applied using PLINK's indep-pairwise with parameter setting of 500Kb window, 50 base pairs sliding window, and r2 threshold of 0.2. Additionally, variants with MAF less than 1% were filtered out. This resulted in a total of 170K variants included in step 1 Imputed genetic dosages were used for step 2 of SAIGE. Variants were only included in the GWAS if they had an imputation quality > 0.3 and a minor allele count (MAC) > 20 within the relevant HARE-defined ancestry group. Analyses were adjusted for age, sex, and 10 ancestry-specific genetic principal components.

Post-GWAS quality control

GWAS results were filtered using a custom R script loosely based on EasyQC.[12] Sanity checks were implemented to remove variants with missing values for major summary statistics (effect size, standard error, etc) or with unreasonable values (pvals or allele frequencies with values >1 or <0). Additionally, variants were removed that were monomorphic, poorly imputed (r2<0.3) or very rare (minor allele frequency < 0.0001) in just the subset of individuals included in the GWAS.

Meta-Analysis

Multi-ancestry meta-analysis was performed using the inverse-variance weighted method as implemented in GWAMA.[13] Meta-analysis results then underwent the same quality control procedures as the GWAS results. Imputation quality filters were not implemented however an additional filter was added to exclude variants that were specific to only one HARE-defined ancestry group.

© 2023 Triozzi JL et al. *JAMA Network Open.*

eReferences

1. Gaziano JM, Concato J, Brophy M, et al. Million Veteran Program: A mega-biobank to study genetic influences on health and disease. *J Clin Epidemiol*. Feb 2016;70:214-23. doi:10.1016/j.jclinepi.2015.09.016
2. Klarin D, Damrauer SM, Cho K, et al. Genetics of blood lipids among ~300,000 multi-ethnic participants of the Million Veteran Program. *Nat Genet*. Nov 2018;50(11):1514-1523. doi:10.1038/s41588-018-0222-9
3. Hunter-Zinck H, Shi Y, Li M, et al. Genotyping Array Design and Data Quality Control in the Million Veteran Program. *Am J Hum Genet*. Apr 2 2020;106(4):535-548. doi:10.1016/j.ajhg.2020.03.004
4. Purcell S, Neale B, Todd-Brown K, et al. PLINK: a tool set for whole-genome association and population-based linkage analyses. *Am J Hum Genet*. Sep 2007;81(3):559-75. doi:10.1086/519795
5. Genomes Project C, Auton A, Brooks LD, et al. A global reference for human genetic variation. *Nature*. Oct 1 2015;526(7571):68-74. doi:10.1038/nature15393
6. Delaneau O, Zagury JF, Robinson MR, Marchini JL, Dermitzakis ET. Accurate, scalable and integrative haplotype estimation. *Nat Commun*. Nov 28 2019;10(1):5436. doi:10.1038/s41467-019-13225-y
7. Howie B, Fuchsberger C, Stephens M, Marchini J, Abecasis GR. Fast and accurate genotype imputation in genome-wide association studies through pre-phasing. *Nat Genet*. Jul 22 2012;44(8):955-9. doi:10.1038/ng.2354
8. Fang H, Hui Q, Lynch J, et al. Harmonizing Genetic Ancestry and Self-identified Race/Ethnicity in Genome-wide Association Studies. *Am J Hum Genet*. Oct 3 2019;105(4):763-772. doi:10.1016/j.ajhg.2019.08.012
9. Song RJ, Ho YL, Schubert P, et al. Phenome-wide association of 1809 phenotypes and COVID-19 disease progression in the Veterans Health Administration Million Veteran Program. *PLoS One*. 2021;16(5):e0251651. doi:10.1371/journal.pone.0251651
10. Denny JC, Ritchie MD, Basford MA, et al. PheWAS: demonstrating the feasibility of a phenome-wide scan to discover gene-disease associations. *Bioinformatics*. May 1 2010;26(9):1205-10. doi:10.1093/bioinformatics/btq126
11. Zhou W, Nielsen JB, Fritsche LG, et al. Efficiently controlling for case-control imbalance and sample relatedness in large-scale genetic association studies. *Nat Genet*. Sep 2018;50(9):1335-1341. doi:10.1038/s41588-018-0184-y
12. Winkler TW, Day FR, Croteau-Chonka DC, et al. Quality control and conduct of genome-wide association meta-analyses. *Nat Protoc*. May 2014;9(5):1192-212. doi:10.1038/nprot.2014.071
13. Magi R, Morris AP. GWAMA: software for genome-wide association meta-analysis. *BMC Bioinformatics*. May 28 2010;11:288. doi:10.1186/1471-2105-11-288

© 2023 Triozzi JL et al. *JAMA Network Open.*
