# Supplementary material for: Mendelian Randomization Analysis of Genetic Proxies of Thiazide Diuretics and the Reduction of Kidney Stone Risk
Source: JAMA Netw Open. 2023 Nov 14;6(11):e2343290. doi: 10.1001/jamanetworkopen.2023.43290 (PMC10646726; doi:10.1001/jamanetworkopen.2023.43290)
Supplement: Supplement 2. — Nonauthor Collaborators [file jamanetwopen-e2343290-s002.pdf]

Supplemental Online Content: Nonauthor Collaborators
*First name, last name, and suffix (if applicable) are required and will appear in PubMed.

*Group Name(s): VA Million Veteran Program

*First Name and Middle Initial(s)	*Last Name	*Suffix (eg, Jr, III)	Academic Degrees	Institution	Location (city, state/province, country)	Role or Contribution, eg, chair, principal investigator	Group (if more than 1 Group listed in the byline) and/or Subgroup (eg, Steering Committee)
Sumitra	Muralidhar		Ph.D.	US Department of Veterans Affairs	Washington, DC, USA	Program Director; Co-	MVP Program Office; MVP Executive Committee
Jennifer	Moser		Ph.D.	US Department of Veterans Affairs	Washington, DC, USA	Associate Director, Sci	MVP Program Office
Jennifer E.	Deen		B.S.	US Department of Veterans Affairs	Washington, DC, USA	Associate Director, Co	MVP Program Office
Philip S.	Tsao		Ph.D.	VA Palo Alto Health Care System	Palo Alto, CA, USA	Co-Principal Investigat	MVP Co-Principal Investigators; MVP Executive Committee; MVP Coordinating Centers; MVP Local Site Investigators
J. Michael	Gaziano		M.D., M.P.	VA Boston Healthcare System	Boston, MA, USA	Co-Principal Investigat	MVP Co-Principal Investigators; MVP Executive Committee; MVP Coordinating Centers
Elizabeth	Hauser		Ph.D.	Durham VA Medical Center	Durham, NC, USA	MVP Executive	MVP Executive Committee
Amy	Kilbourne		Ph.D., M.P.	VA HSR&D	Ann Arbor, MI, USA	MVP Executive	MVP Executive Committee
Shiuh-Wen	Luoh		M.D., Ph.D.	VA Portland Health Care System	Portland, OR, USA	MVP Executive	MVP Executive Committee
Michael	Matheny		M.D., M.S.,	VA Tennessee Valley Healthcare Syst	Nashville, TN, USA	MVP Executive	MVP Executive Committee
Dave	Oslin		M.D.	Philadelphia VA Medical Center	Philadelphia, PA, USA	MVP Executive	MVP Executive Committee
Lori	Churby		B.S.	VA Palo Alto Health Care System	Palo Alto, CA, USA	Director, MVP Regulat	MVP Core Operations
Stacey B.	Whitbourne		Ph.D.	VA Boston Healthcare System	Boston, MA, USA	Director, MVP Cohort	MVP Core Operations
Jessica V.	Brewer		M.P.H.	VA Boston Healthcare System	Boston, MA, USA	Director, MVP Recruit	MVP Core Operations
Shahpoor (Alex)	Shayan		M.S.	VA Boston Healthcare System	Boston, MA, USA	Director, MVP Recruit	MVP Core Operations
Luis E.	Selva		Ph.D.	VA Boston Healthcare System	Boston, MA, USA	Executive Director, M	MVP Core Operations
Saiju	Pyarajan		Ph.D.	VA Boston Healthcare System	Boston, MA, USA	Director, Data and Con	MVP Core Operations
Kelly	Cho		M.P.H, Ph.	VA Boston Healthcare System	Boston, MA, USA	Director, MVP Phenom	MVP Core Operations
Scott L.	DuVall		Ph.D.	VA Salt Lake City Health Care System	Salt Lake City, UT, USA	Director, VA Informati	MVP Core Operations
Mary T.	Brophy		M.D., M.P.	VA Boston Healthcare System	Boston, MA, USA	Director, VA Central Bi	MVP Core Operations
Brady	Stephens		M.S.	Canandaigua VA Medical Center	Canandaigua, NY, USA	MVP Information Cent	MVP Coordinating Centers
Todd	Connor		Pharm.D.	New Mexico VA Health Care System	Albuquerque, NM, USA	CSP Clinical Research	MVP Coordinating Centers; Cooperative Studies Program
Dean P.	Argyres		B.S., M.S.	New Mexico VA Health Care System	Albuquerque, NM, USA	CSP Clinical Research	MVP Coordinating Centers; Cooperative Studies Program
Tim	Assimes		M.D.	VA Palo Alto Health Care System	Palo Alto, CA, USA	Co-Chair	MVP Publications and Presentations Committee
Adriana	Hung		M.D.	VA Tennessee Valley Healthcare Syst	Nashville, TN, USA	Co-Chair; Local Site Inv	MVP Publications and Presentations Committee; MVP Local Site Investigators
Henry	Kranzler		M.D.	Philadelphia VA Medical Center	Philadelphia, PA, USA	Co-Chair	MVP Publications and Presentations Committee
Samuel	Aguayo		M.D.	Phoenix VA Health Care System	Phoenix, AZ, USA	Local Site Investigator	MVP Local Site Investigators
Sunil	Ahuja		M.D.	South Texas Veterans Health Care Sy	San Antonio, TX, USA	Local Site Investigator	MVP Local Site Investigators
Kathrina	Alexander		M.D.	Veterans Health Care System of the	Fayetteville, AR, USA	Local Site Investigator	MVP Local Site Investigators
Xiao M.	Androulakis		M.D.	Columbia VA Health Care System	Columbia, SC, USA	Local Site Investigator	MVP Local Site Investigators
Prakash	Balasubramanian		M.D.	William S. Middleton Memorial Vete	Madison, WI, USA	Local Site Investigator	MVP Local Site Investigators
Zuhair	Ballas		M.D.	Iowa City VA Health Care System	Iowa City, IA, USA	Local Site Investigator	MVP Local Site Investigators
Jean	Beckham		Ph.D.	Durham VA Medical Center	Durham, NC, USA	Local Site Investigator	MVP Local Site Investigators
Sujata	Bhushan		M.D.	VA North Texas Health Care System	Dallas, TX, USA	Local Site Investigator	MVP Local Site Investigators
Edward	Boyko		M.D.	VA Puget Sound Health Care System	Seattle, WA, USA	Local Site Investigator	MVP Local Site Investigators
David	Cohen		M.D.	Portland VA Medical Center	Portland, OR, USA	Local Site Investigator	MVP Local Site Investigators
Louis	Dellitalia		M.D.	Birmingham VA Medical Center	Birmingham, AL, USA	Local Site Investigator	MVP Local Site Investigators
L. Christine	Faulk		M.D.	Robert J. Dole VA Medical Center	Wichita, KS, USA	Local Site Investigator	MVP Local Site Investigators
Joseph	Fayad		M.D.	VA Southern Nevada Healthcare Syst	North Las Vegas, NV, USA	Local Site Investigator	MVP Local Site Investigators
Daryl	Fujii		Ph.D.	VA Pacific Islands Health Care System	Honolulu, HI, USA	Local Site Investigator	MVP Local Site Investigators
Saib	Gappy		M.D.	John D. Dingell VA Medical Center	Detroit, MI, USA	Local Site Investigator	MVP Local Site Investigators
Frank	Gesek		Ph.D.	White River Junction VA Medical Cen	White River Junction, VT,	Local Site Investigator	MVP Local Site Investigators
Jennifer	Greco		M.D.	Sioux Falls VA Health Care System	Sioux Falls, SD, USA	Local Site Investigator	MVP Local Site Investigators
Michael	Godschalk		M.D.	Richmond VA Medical Center	Richmond, VA, USA	Local Site Investigator	MVP Local Site Investigators
Todd W.	Gress		M.D., Ph.D.	Hershel “Woody” Williams VA Medic	Huntington, WV, USA	Local Site Investigator	MVP Local Site Investigators
Samir	Gupta		M.D., M.S.	VA San Diego Healthcare System	San Diego, CA, USA	Local Site Investigator	MVP Local Site Investigators
Salvador	Gutierrez		M.D.	Edward Hines, Jr. VA Medical Center	Hines, IL, USA	Local Site Investigator	MVP Local Site Investigators
John	Harley		M.D., Ph.D.	Cincinnati VA Medical Center	Cincinnati, OH, USA	Local Site Investigator	MVP Local Site Investigators
Kimberly	Hammer		Ph.D.	Fargo VA Health Care System	Fargo, ND, USA	Local Site Investigator	MVP Local Site Investigators
Mark	Hamner		M.D.	Ralph H. Johnson VA Medical Center	Charleston, SC, USA	Local Site Investigator	MVP Local Site Investigators
Robin	Hurley		M.D.	W.G. (Bill) Hefner VA Medical Center	Salisbury, NC, USA	Local Site Investigator	MVP Local Site Investigators

Supplemental Online Content: Nonauthor Collaborators
*First name, last name, and suffix (if applicable) are required and will appear in PubMed.

***First Name and Middle Initial(s)**	***Last Name**	***Suffix (eg, Jr, III)**	Academic Degrees	Institution	Location (city, state/province, country)	Role or Contribution, eg, chair, principal investigator	Group (if more than 1 Group listed in the byline) and/or Subgroup (eg, Steering Committee)
Pran	Iruvanti		D.O., Ph.D.	Hampton VA Medical Center	Hampton, VA, USA	Local Site Investigator	MVP Local Site Investigators
Frank	Jacono		M.D.	VA Northeast Ohio Healthcare Systen	Cleveland, OH, USA	Local Site Investigator	MVP Local Site Investigators
Darshana	Jhala		M.D.	Philadelphia VA Medical Center	Philadelphia, PA, USA	Local Site Investigator	MVP Local Site Investigators
Scott	Kinlay		M.B.B.S., P	VA Boston Healthcare System	Boston, MA, USA	Local Site Investigator	MVP Local Site Investigators
Jon	Klein		M.D., Ph.D.	Louisville VA Medical Center	Louisville, KY, USA	Local Site Investigator	MVP Local Site Investigators
Michael	Landry		Ph.D.	Southeast Louisiana Veterans Health	New Orleans, LA, USA	Local Site Investigator	MVP Local Site Investigators
Peter	Liang		M.D., M.P.	VA New York Harbor Healthcare Syst	New York, NY, USA	Local Site Investigator	MVP Local Site Investigators
Suthat	Liangpunsakul		M.D., M.P.	Richard Roudebush VA Medical Cent	Indianapolis, IN, USA	Local Site Investigator	MVP Local Site Investigators
Jack	Lichy		M.D., Ph.D.	Washington DC VA Medical Center	Washington, D. C., USA	Local Site Investigator	MVP Local Site Investigators
C. Scott	Mahan		M.D.	Charles George VA Medical Center	Asheville, NC, USA	Local Site Investigator	MVP Local Site Investigators
Ronnie	Marrache		M.D.	VA Maine Healthcare System	Augusta, ME, USA	Local Site Investigator	MVP Local Site Investigators
Stephen	Mastorides		M.D.	James A. Haley Veterans’ Hospital	Tampa, FL, USA	Local Site Investigator	MVP Local Site Investigators
Elisabeth	Mates		M.D., Ph.D.	VA Sierra Nevada Health Care System	Reno, NV, USA	Local Site Investigator	MVP Local Site Investigators
Kristin	Mattocks		Ph.D., M.P.	Central Western Massachusetts Heal	Leeds, MA, USA	Local Site Investigator	MVP Local Site Investigators
Paul	Meyer		M.D., Ph.D.	Southern Arizona VA Health Care Sys	Tucson, AZ, USA	Local Site Investigator	MVP Local Site Investigators
Jonathan	Moorman		M.D., Ph.D.	James H. Quillen VA Medical Center	Mountain Home, TN, USA	Local Site Investigator	MVP Local Site Investigators
Timothy	Morgan		M.D.	VA Long Beach Healthcare System	Long Beach, CA, USA	Local Site Investigator	MVP Local Site Investigators
Maureen	Murdoch		M.D., M.P.	Minneapolis VA Health Care System	Minneapolis, MN, USA	Local Site Investigator	MVP Local Site Investigators
James	Norton		Ph.D.	VA Health Care Upstate New York	Albany, NY, USA	Local Site Investigator	MVP Local Site Investigators
Olaoluwa	Okusaga		M.D.	Michael E. DeBakey VA Medical Cent	Houston, TX, USA	Local Site Investigator	MVP Local Site Investigators
Kris Ann	Oursler		M.D.	Salem VA Medical Center	Salem, VA, USA	Local Site Investigator	MVP Local Site Investigators
Ana	Palacio		M.D., M.P.	Miami VA Health Care System	Miami, FL, USA	Local Site Investigator	MVP Local Site Investigators
Samuel	Poon		M.D.	Manchester VA Medical Center	Manchester, NH, USA	Local Site Investigator	MVP Local Site Investigators
Emily	Potter		Pharm.D.	VA Eastern Kansas Health Care Syste	Leavenworth, KS, USA	Local Site Investigator	MVP Local Site Investigators
Michael	Rauchman		M.D.	St. Louis VA Health Care System	St. Louis, MO, USA	Local Site Investigator	MVP Local Site Investigators
Richard	Servatius		Ph.D.	Syracuse VA Medical Center	Syracuse, NY, USA	Local Site Investigator	MVP Local Site Investigators
Satish	Sharma		M.D.	Providence VA Medical Center	Providence, RI, USA	Local Site Investigator	MVP Local Site Investigators
River	Smith		Ph.D.	Eastern Oklahoma VA Health Care Sy	Muskogee, OK, USA	Local Site Investigator	MVP Local Site Investigators
Peruvemba	Sriram		M.D.	N. FL/S. GA Veterans Health System	Gainesville, FL, USA	Local Site Investigator	MVP Local Site Investigators
Patrick	Strollo Jr.		M.D.	VA Pittsburgh Health Care System	Pittsburgh, PA, USA	Local Site Investigator	MVP Local Site Investigators
Neeraj	Tandon		M.D.	Overton Brooks VA Medical Center	Shreveport, LA, USA	Local Site Investigator	MVP Local Site Investigators
Gerardo	Villareal		M.D.	New Mexico VA Health Care System	Albuquerque, NM, USA	Local Site Investigator	MVP Local Site Investigators
Agnes	Wallbom		M.D., M.S.	VA Greater Los Angeles Health Care S	Los Angeles, CA, USA	Local Site Investigator	MVP Local Site Investigators
Jessica	Walsh		M.D.	VA Salt Lake City Health Care System	Salt Lake City, UT, USA	Local Site Investigator	MVP Local Site Investigators
John	Wells		Ph.D.	Edith Nourse Rogers Memorial Veter	Bedford, MA, USA	Local Site Investigator	MVP Local Site Investigators
Jeffrey	Whittle		M.D., M.P.	Clement J. Zablocki VA Medical Cent	Milwaukee, WI, USA	Local Site Investigator	MVP Local Site Investigators
Mary	Whooley		M.D.	San Francisco VA Health Care System	San Francisco, CA, USA	Local Site Investigator	MVP Local Site Investigators
Allison E.	Williams		N.D., Ph.D.,	Bay Pines VA Healthcare System	Bay Pines, FL, USA	Local Site Investigator	MVP Local Site Investigators
Peter	Wilson		M.D.	Atlanta VA Medical Center	Decatur, GA, USA	Local Site Investigator	MVP Local Site Investigators
Junzhe	Xu		M.D.	VA Western New York Healthcare Sys	Buffalo, NY, USA	Local Site Investigator	MVP Local Site Investigators
Shing Shing	Yeh		Ph.D., M.D.	Northport VA Medical Center	Northport, NY, USA	Local Site Investigator	MVP Local Site Investigators
