# Supplementary material for: Mendelian Randomization Analysis of Genetic Proxies of Thiazide Diuretics and the Reduction of Kidney Stone Risk
Source: JAMA Netw Open. 2023 Nov 14;6(11):e2343290. doi: 10.1001/jamanetworkopen.2023.43290 (PMC10646726; doi:10.1001/jamanetworkopen.2023.43290)
Supplement: Supplement 3. — Data Sharing Statement [file jamanetwopen-e2343290-s003.pdf]

Data Sharing Statement

Triozzi. Mendelian Randomization Analysis of Genetic Proxies of Thiazide Diuretics and the Reduction of Kidney Stone Risk. *JAMA Netw Open*. Published November 14, 2023. doi:10.1001/jamanetworkopen.2023.43290

Data
Data available: No

Additional Information
Explanation for why data not available: All instrumental variables and their harmonization with outcome summary statistics are provided in the Supplemental Content. ICBP summary data can be assessed through request to ICBP steering committee. MVP summary statistics are available from the database of Genotypes and Phenotypes through dbGaP accession number phs002453. Pan-UKBB project summary statistics are available at https://pan.ukbb.broadinstitute.org/. FinnGen summary statistics are available at https://www.finngen.fi/en/access_results.
